# Antioxidant Activity of *Myrtus communis* L. and *Myrtus nivellei* Batt. & Trab. Extracts: A Brief Review

**DOI:** 10.3390/medicines5030089

**Published:** 2018-08-11

**Authors:** Aicha Hennia, Maria Graça Miguel, Said Nemmiche

**Affiliations:** 1 Department of Agronomy, Faculty of Nature and Life Sciences, University of Mostaganem, BP 188/227, Mostaganem 27000, Algeria; a.hennia@gmail.com; 2 Departamento de Química e Farmácia, Faculdade de Ciências e Tecnologia, Universidade do Algarve, MeditBio, Campus de Gambelas, 8005-139 Faro, Portugal; 3 Department of Biology, Faculty of Nature and Life Sciences, University of Mostaganem, BP 188/227, Mostaganem 27000, Algeria; snemiche@hotmail.com

**Keywords:** Anti-inflammatory, berries, leaves, galloyl derivatives, flavonol derivatives, anthocyanins, myrtucommulone

## Abstract

*Myrtus communis* L. (myrtle) and *Myrtus nivellei* Batt. & Trab. (Saharan myrtle) have been used in folk medicine for alleviating some ailments. *M. communis* is largely distributed in the Mediterranean Basin, whereas *M. nivellei* is confined in specific zones of the central Saharan mountains. The chemical composition and antioxidant activity of berry and leaf extracts isolated from myrtle are deeply documented, whereas those isolated from Saharan myrtle extracts are less studied. In both species, the major groups of constituents include gallic acid derivatives, flavonols, flavonol derivatives, and hydroxybenzoic acids. In coloured berries, anthocyanins are also present. In *M. nivellei* extracts are reported for some compounds not described in *M. communis* so far: 2-hydroxy-1,8-cineole-β-d-glucopyranoside, 2-hydroxy-1,8-cineole 2-*O*-α-l-arabinofuranosyl (1→6)-β-d-glucopyranoside, rugosin A, and rugosin B. Berries and leaves extracts of both species had antioxidant activity. Comparative studies of the antioxidant activity between leaf and berry myrtle extracts revealed that leaf extracts are best antioxidants, which can be assigned to the galloyl derivatives, flavonols, and flavonols derivatives, although the ratio of these groups of compounds might also have an important role in the antioxidant activity. The anthocyanins present in myrtle berries seem to possess weak antioxidant activity. The antioxidant activity of sample extracts depended on various factors: harvesting time, storage, extraction solvent, extraction type, and plant part used, among other factors. Leaf extracts of myrtle revealed to possess anti-inflammatory activity in several models used. This property has been attributed either to the flavonoids and/or hydrolysable tannins, nevertheless nonprenylated acylphloroglucinols (e.g., myrtucommulone and semimyrtucommulone) have also revealed a remarkable role in that activity. The biological activities of myrtle extracts found so far may direct its use towards for stabilizing complex lipid systems, as prebiotic in food formulations, and as novel therapeutic for the management of inflammation.

## 1. Introduction

Myrtaceae is a family of woody flowering plants that encompasses around 5500 species, classified in 144 genera, and 17 tribes. Within Myrtaceae, the tribe Myrteae represents half of the family’s biodiversity with 51 genera and about 2500 species mostly restricted to the Neotropics, though 15 genera and about 450 species are found in other continents, such as Southeast Asia, Northeast Australia, and the Pacific islands, including New Caledonia and New Zealand. The genus *Myrtus* is the sole found in European/Northern African, Asia, particularly in the Mediterranean region of southern Europe as far west as Macaronesia (Madeira and the Azores), the Saharan mountains and as far east as western Asia (Iran and Afghanistan) [1,2,3].

Two species can be found in the genus *Myrtus*: *Myrtus communis* L. and *Myrtus nivellei* Batt. & Trab. The latter is endemic to the central Saharan mountains growing in rocky and sandy wades and gorges, at high elevations, above 1400 m. The former can be found in the Mediterranean Basin, Macaronesia, Iran, and Afghanistan, particularly at elevations not exceeding c.a. 500 m a.s.l. [1]. Both species are shrubs with rough bark, opposite leaves, white flowers that are star-like (5–9 petals), and white, purple, blue, or even black berries. They differ in the following morphological characteristics: the leaves of *M. nivellei* are linear-lanceolate (4–5 cm in length) and narrower (6–8 mm) than the *M. communis* ones, which are ovate-lanceolate (2–5 cm long) and wider (10–20 mm); the fruits of *M. communis* are ellipsoid to subglobose, pyriform, elongated, or flat (7–9 mm length), whereas those of *M. nivellei* are globose and smaller (4–5 mm) [1,4,5,6]. *M. communis* grows to 0.5–3 m in height, while *M. nivelli* grows to 1–2 m in height [1].

Different parts (berries, branches, and leaves) of *M. communis* (myrtle) have been used in folk medicine for treating diarrhoea, peptic ulcers, haemorrhoids, inflammation, uterine bleeding, headache, palpitation, leucorrhoea, urethritis, epistaxis, conjunctivitis, excessive perspiration, and pulmonary and skin diseases [4,7]. Only few studies have reported a sedative effect of myrtle, as a anxiolytic and muscle relaxant without anticonvulsivant activity [8,9].

Myrtle leaves have been used for healing wounds or disorders of the digestive and urinary systems due to their astringent, tonic, and antiseptic properties [4,10]. From leaves is also possible to extract essential oils that have been used as anti-septic, anti-catarrhal, and to treat chest ailments, ulcers, and hemorrhoids [4,10,11,12,13].

Although the berries decoctions had been used to bathe newborns with reddened skin, and the decoctions of leaves and berries in sore washing, the most is used to produce the characteristic myrtle liqueur obtained by hydro-alcoholic infusion of the berries [4,14,15].

The biological properties assigned to diverse organs (leaves and berries) of myrtle can be due to diverse compounds such as volatile compounds or essential oils (terpenoids, particularly α-pinene, 1,8-cineole, geranyl acetate, and linalool), flavonoids (quercetin, catechin and myricetin derivatives, and anthocyanins), coumarins, oligomeric nonprenylated acylphloroglucinol compounds (myrtucommulone A and B and semimyrtucommulone), galloyl-glucosides, ellagitannins, galloyl-quinic acids, caffeic, gallic and ellagic acids, fatty acids (linoleic, palmitic, oleic, and stearic acids) in diverse organs [4]. Table 1 shows examples of biological properties assigned to *Myrtus communis*.

*M. nivellei* (Saharan myrtle) leaves in infusions are used against intestinal diseases (diarrhoea), fever, diabetes, and added to barley wafers is employed against blennorrhoea [16,17,18]. The crushed leaves added to oil or butter ointment has been used in the treatment of dermatosis and for hair and body care [16,17,19]. The decoction of leaves mixed with goat milk and heated on charcoal has been used for liver disorders by nomad Algerians of Tassili region [20]. The leaf infusion is used in this region as a common beverage, instead of green tea [20]. Berries are consumed either fresh or dried to treat mouth canker sores [19].

The chemical composition of Saharan myrtle is less studied than that of myrtle. The main constituents reported include volatile essential oils [16,21], phenols (flavonoids, anthocyanins, and tannins), norterpenoids [19,20,21,22].

The present review will focus on the antioxidant and anti-inflammatory activities of *M. communis* and *M. nivellei* in which the chemical composition is discriminated.

### 2. *Myrtus communis*: Berries

All works regarding the antioxidant activity of berry extracts reported their capacity for preventing lipid peroxidation or capacity for scavenging free radicals. In the majority of cases, the evaluation was done *in vitro* as can be read below. The presentation of results was diverse, hampering many times the comparison of the results. In addition, in those cases where the identification of compounds was done, very few works correlate the contribution of each phenol compound on the antioxidant activity. Factors such as type of extraction, solvents, maturation stage, storage conditions, variety, different parts of the fruit, different organs that could influence the chemical profile, and antioxidant activity of myrtle extracts were evaluated by diverse teams, as can be read below. Sanjust et al. [96] had previously reviewed the antioxidant activity of myrtle liqueur along with other Mediterranean shrubs *Arbutus unedo*, *A. andrachne*, *Capparis spinosa*, *Opuntia ficus-indica*, *Rosa canina*, *Rosmarinus officinalis*, and *Rubus fruticosus*.

#### 2.1. Myrtle Liqueur

One of the most applications of berries is to produce the myrtle liqueur obtained by hydro-alcoholic infusion of the berries. For this reason, there are some works evaluating the antioxidant of myrtle liqueur as described beneath.

Previously, Alamanni and Cossu [97] not only reported the antioxidant activity measured to the ability for scavenging DPPH (2,2-diphenyl-1-picrylhydrazyl) free radicals, conductimetric method, and the linoleic acid test of liquors made from berries and leaves *Myrtus communis* L. (eight industrially-prepared and three laboratory-prepared samples), as also correlated with the amounts of phenols in samples, although not identifying such metabolites. Simultaneously, the authors compare the activities of samples with some red wines and synthetic antioxidant standards (Butylated hydroxytoluene (BHT) and butylated hydroxyanisole (BHA)) and a natural standard (catechin). The results showed that samples had capacity for scavenging free radicals and had also higher antioxidant index and induction time in the conductimetric test. In both tests, the authors found a correlation between the activities and the phenol concentration of samples. This correlation was low in the linoleic acid test. For the same concentration of phenols, berry liquors showed higher protection against fatty acid oxidation and red wines presented better protection than liquors [97]. Concerning the capacity for scavenging free radicals, both berry and leaf liquours had higher activity than red wines. The amounts of phenols ranged from 0.17 to 1.47 g/L.

The correlation between phenol and anthocyanins amounts and antioxidant activity was poorly significant in the results obtained by Vacca et al. [98] when studying the effect of type and time of storage of myrtle liqueur on the accumulation of phenols and anthocyanins as well as in the capacity for scavenging the DPPH free radicals. The antioxidant activity of myrtle liqueur decreased over the time, with a loss of about 20% by the end of storage in opened bottles, in contrast to the unopened bottles in which the activity practically remained constant, even after one year of storage. Either in open or unopened bottles, the free and combined anthocyanins decreased, nevertheless faster and more intensively in last ones [98]. Montoro et al. [15] showed that berry extracts of *M. communis* prepared for liqueur recipe were not stable during one year of storage, being flavonoids and, particularly, anthocyanins the most unstable compounds. Their results have even allowed to state that the use of extracts should not exceed three months. During this period, the antioxidant activity (scavenging 2,2′-azino-*bis*-3-ethylbenzthiazoline-6-sulphonic acid (ABTS) free radicals capacity) would be preserved. In addition, the authors also identified by high performance liquid chromatography (HPLC) coupled with electrospray mass spectrometry (ESMS) the flavonoids and anthocyanins (Table 2) present in the extract, along with their quantification by HPLC coupled with Ultraviolet/visible detection (UV/Vis), over the storage period. For obtaining a final product of myrtle liqueur with the traditional characteristics, the starting material should be fresh or lyophilised and the extraction should be only the maceration, excluding other procedures such as the ultrasonic extraction. The initial extract had capacity for scavenging the ABTS free radical that increased after 3 months of storage. Such is coincident with the hydrolysis of the flavon glycosides, that is, higher accumulation of myricetin, which leads to an additional hydroxyl group able to participate in the redox reaction. However, after 8 months of storage the antioxidant activity was stable and similar to the fresh extract, despite significant decrease of anthocyanins but higher amounts of myricetin [15].

For myrtle liqueur production, berries must be processed immediately after harvest to prevent quality loss such as decay and development of off flavours. For this reason, liqueur industries immediately process the berries and store the hydro-alcoholic extracts. Nevertheless, some anthocyanins decreased over time and the extract loses the initial characteristics [15]. Another approach is to store berries in cold conditions during a defined period, or even freeze them, however, some studies have also concluded that these procedures are not free of undesirable effects, namely in the alteration of fruit composition and, therefore, in the liqueur quality [99]. According to Fadda et al. [14], oxygen-enriched atmospheres have been successfully used to retain the quality of stored fruit and vegetables. For this reason, they proposed to evaluate the effect of different high oxygen treatments on physicochemical quality of myrtle berries and their corresponding hydro-alcoholic extracts used for the preparation of the liqueur. Oxygen treatments induced an increase of total phenols in stored berries, for example, berries held at 80% O_2_ had higher total phenols than 60% O_2_ and control fruit after 10 and 20 days of storage, nevertheless practically without differences after 30 days. Anthocyanins’ contents were also higher in those berries submitted to higher levels of O_2_, particularly after 10–20 days of storage. The capacity for scavenging DPPH free radicals was also higher in those samples held at 80% O_2_ after 20 days, and decreasing afterwards. Oxygen treatments did not influence the ability of samples for quenching ABTS free radicals. Such results allowed concluding that myrtle fruit held at oxygen concentration between 60% and 80%, for 20 days at 2 °C, preserve quality with higher phenolic and anthocyanin concentration [14].

For producing liqueur of myrtle berries, Zam et al. [100] studied the effect of different extracts from Syrian wild myrtle berries and their mixtures with cloves and cinnamon added as flavours on the phenolic fractions and antioxidant activity. Hydroalcoholic mixtures in the range of 50–80% and a maceration process for 5 months were used. Concerning the total polyphenols the authors observed that the extraction mixture with higher percentage of water extracted higher amount of phenols than the extraction mixture with higher percentage of ethanol. According to the authors, such may be attributed to several factors: high concentrations of ethanol will denature proteins which prevent the dissolution of phenols, and low levels of ethanol in the extraction will permit to access easily into cells and dissociate the complex phenolic compounds bound to proteins and polysaccharides into the cell walls [100]. Only the addition of clove originates higher amounts of polyphenols in the samples. During the maceration period there was an increase of the amounts of total polyphenols being also dependent on the concentrations of ethanol. The highest phenol concentration was observed in the hydroalcoholic extract (50:50) with cloves, and after 60 days of maceration (7.82 g/100 g, dry weight). The antioxidant activity was also measured through the ability of samples for scavenging the DPPH free radicals. All samples presented variable antioxidant activity depending on the type of extract, concentration of alcohol, and time of maceration. The sample extracted with the hydroalcoholic (50:50) solution and after 60 days of maceration in myrtle berries:cinnamon:clove and myrtle berries:clove mixtures had the best capacity for scavenging the DPPH free radicals (80.95% and 80.02%, respectively). The results indicate a positive correlation between the total content of polyphenols and the antioxidant activity. At the end, the authors [100] concluded that the extract obtained with the mix ethanol/water (50:50) with cloves was the most adequate for providing the better characteristics for liqueur preparation.

The extraction efficiency in the preparation of myrtle liqueur was also evaluated by Snoussi et al. [101]. In order to achieve the objective, the authors assayed different alcohol–water mixtures (60–90% ethanol), for 40 days. Flavonoids and anthocyanins were identified (Table 2) by HPLC/ESMS and quantified by HPLC/UV/Vis. The antioxidant activity was assayed using the method of DPPH. The results showed that higher amounts of total polyphenols were obtained in the extract with 80% ethanol, coinciding with the highest capacity for scavenging the DPPH free radicals (87.5%). The minimum activity was found in the extract with 60% ethanol (65.0%). The best phenol extraction with mixtures of solvents with higher proportions of alcohol contrasts with those reported by [100]. In addition, during the maceration period, a reduction in the concentrations of the identified compounds was observed. For example, the content of the major constituent of the extract, malvidin-3-*O*-glucoside, decreased with a loss of 30–40% after 40 days of storage. According to the authors [101], such loss can be attributed to the degradation, combination with other compounds providing more stable polymeric pigments. Differences in the antioxidant activity can be attributed not only to the amounts of phenols but also to their structures, according to the authors, nevertheless none study was performed by them to clarify this statement [101].

Tuberoso et al. [102] used three methods (capacity for scavenging the DPPH and ABTS free radicals, and ferric reducing antioxidant power (FRAP)) for evaluating the antioxidant activity of liqueurs obtained by cold maceration of myrtle berries and compared the results with other two typical food products from the Mediterranean area (red wines Cannonau and strawberry-tree honey). Simultaneously, the authors [102] proceeded in identifying the secondary metabolites by LC-MS/MS (liquid chromatography tandem mass spectrometry) and also dosing them by HPLC-DAD (High-Performance Liquid Chromatography with Diode-Array Detection). Cannonau wine and myrtle liqueur showed high levels of total polyphenols (1978 and 1741 mg gallic acid equivalent/L, respectively). A positive correlation between the results of FRAP, ABTS, and DPPH assays and total polyphenols were observed by the authors [102]. Despite this correlation, myrtle liqueur, wines Cannonau and strawberry-tree honey presented different antioxidant activities. The authors did not determine the antioxidant activity of each phenol compound identified in samples and, therefore, they were not able to attribute those activities to any compound, nevertheless they suggested that the different types of phenols could be responsible for the differences observed. Only some examples pointed out by the authors [102]: myrtle liqueur was characterized by myricetin-3-*O*-arabinoside (not detected in Cannonau wine), quercetin-3-*O*-glucuronide and kaempferol-3-*O*-glucoside were detected only in Cannonau wine; strawberry-tree honey showed homogentisic acid as the most prominent phenolic compound, which was absent in myrtle liqueur and Cannonau wine. The antioxidant activities of myrtle liqueur found by the authors were as follows: 26.7 mmol Fe^2+^/L, 9.3 mmol TEAC/L, and 11.5 mmol TEAC/L for the FRAP, DPPH, and ABTS assays, respectively [102].

The antioxidant capacity of myrtle liqueur obtained from white myrtle berry was also determined by Serreli et al. [103]. The identification and quantification of the phenols were followed by LC-MS/MS and HPLC-DAD, respectively. The antioxidant activity was determined through ABTS, DPPH, FRAP, and CUPRAC (CUPric Reducing Antioxidant Capacity) assays. The constituents of the volatile fraction of liqueur samples were also identified by gas chromatography and mass spectrometry (GC-MS) and quantified by GC-FID (GC-flame ionization detector) after headspace solid-phase microextraction (HS-SPME) and liquid–liquid extraction (LLE). According to the results obtained, lower amounts of total polyphenols were found by the authors in the white myrtle berry liqueur (636.3 mg gallic acid equivalent/L), when they compared their results with those of other authors that used purple berries (1741 mg gallic acid equivalent/L) [102]. Despite this difference, white myrtle berry liqueur did not exhibit poorer antioxidant activity than purple myrtle berry liqueur [102]. The antioxidant activities of white myrtle liqueur found by the authors were as follows: 30.21 mmol Fe^2+^/L, 3.72 mmol TEAC/L, and 11.66 mmol TEAC/L for the FRAP, DPPH, and ABTS assays, respectively [103]. The antioxidant activity measured through the CUPRAC method was 11.30 mmol Fe^2+^/L. Once again, the contribution of each compound on the antioxidant activity was not made by the authors; however they attributed the similar or even better activity of their samples (liqueurs obtained from white myrtle berries) to the highest amounts of gallic acid and their derivatives (Table 2), although other groups of polyphenols are also present in the liqueur samples (Table 2). In the volatile fraction, terpenes predominated in white myrtle berry liqueurs, nevertheless other ones could also be detected such as 4-hydroxybenzyl alcohol, ethyl 4-hydroxybenzoate, 4-hydroxybenzoic acid, vanillic acid, and ethyl vanillate, all of them shikimic acid pathway derivatives, which could also contribute to the antioxidant activity [103].

#### 2.2. Antioxidant Activity of Berry Extracts

The antioxidant activities of berry extracts were also performed in diverse works. For example, [104] reported that the methanolic extracts of eight accessions of Turkish myrtle fruits had capacity for scavenging DPPH free radicals as well as inhibiting linoleic acid oxidation measured through the method of β-carotene-bleaching test, although with different strenght. In the DPPH assay, the IC_50_ values found ranged from 2.34 μg/mL, not significantly different to that of the reference α-tocopherol, and 8.24 μg/mL. In what concerns, the ability for preventing linoleic acid oxidation, the percentages of inhibition were always above 80%, nevertheless lower than the percentage registered for reference (α-tocopherol); that was 96.31%. The effect of extracting solvents on the total phenolic content, antioxidant, and antiradical activity of extracts of myrtle berry, collected in different places of Turkey, was also studied by Polat et al. [105]. The antioxidant activity, evaluated through the phosphomolybdenum spectrophotometric method, revealed that methanolic extract presented the highest antioxidant value (241.533 mg ascorbic acid equivalents/g dry extract). Overall, the phenol content ranged from 39.933 to 207.4 mg gallic acid equivalent/g. The capacity for scavenging DPPH free radicals ranged between 6.73% and 65.6%.

The antioxidant activity of extracts obtained from white and dark blue Tunisian berries was evaluated by [106]. The chemical composition was also evaluated by the authors: essential oils, fatty acids, and anthocyanins. Dark blue fruits produced extracts with higher antioxidant activity, measured through the capacity of scavenging the DPPH free radicals and ferric reducing antioxidant power (IC_50_ = 2.1 mg/mL and 2.6 mmol Fe^2+^/g, respectively) than white fruits (2.8 mg/mL and 2.1 mmol Fe^2+^/g, respectively). These results are different to those previously reported [103], because these authors did not observe lower activity in liqueurs made with white berries. However, and as expected, in both cases the levels of anthocyanins in white berries is lower than in red or dark purple berries (Table 2). Total polyphenols, flavonoids, and flavonols were higher in coloured berries than in white ones. The authors attributed the antioxidant capacity variation between the two myrtle types to their different phenolic contents [106].

Other factors that can influence the phenol/anthocyanin content and antioxidant activity of myrtle extracts have been studied by diverse research teams: variety [107,108], part of the berry [107], maturation [109], method of extraction, and type of solvent [110,111,112].

The antioxidant activity of myrtle berry extracts prepared with solvents at diverse polarities (water, alcohol, and ethyl acetate) was evaluated by Tuberoso et al. [110] for the first time. The authors evaluated the capacity of those extracts for scavenging the DPPH free radicals and their capacity to protect biological molecules using the cholesterol and LDL (low density lipoproteins) oxidation assays. In the same work, the identification of phenolic compounds was assigned by HPLC-DAD and HPLC-MS/MS (Table 2). The ethyl acetate extract had the highest capacity for inhibiting the reduction of polyunsaturated fatty acids and cholesterol, and the increase of their oxidative products [110]. Moreover, higher amounts of phenols were found in the aqueous and ethyl acetate extracts which coincided with the highest antioxidant activity, meaning a high correlation between the concentration of phenols and the antioxidant activity. However, the contribution of each phenol compound on the antioxidant activity, independent on the method used, was not clarified. Besides the protective effect of myricetin-3-*O*-galactoside and myricetin-3-*O*-rhamnoside on cholesterol and human LDL oxidation, since they are generally considered excellent in inhibiting free radical and lipid peroxidation, other compounds might have contributed to the best activity of the ethyl acetate extract, such as gallic acid derivatives [110].

Methanolic extracts of whole fruit, seed and pericarp of *M. communis* var. *italica* were analysed in terms of total phenols, flavonoids, anthocyanins, and antioxidant activity (DPPH, β-carotene-linoleic acid bleaching and reducing power assays) [107]. The total phenol and tannins contents varied among different parts of myrtle fruit; seed extract had higher total phenol and tannin contents than whole fruit, while total flavonoid contents were higher in pericarp. The compounds identified by the authors are depicted in Table 2. Methanolic seed extracts showed higher scavenging ability on DPPH radicals (IC_50_ = 8 μg/mL) than whole fruit (IC_50_ = 136 μg/mL) and pericarp (IC_50_ = 196 μg/mL), which can be attributed to the highest levels of hydrolysable tannins [107]. Seed methanolic extract also showed a higher ability to prevent the bleaching of β-carotene (IC_50_ = 70 μg/mL) than whole fruit (IC_50_ = 78 μg/mL) and pericarp (IC_50_ = 150 μg/mL). The reducing power of seed methanolic extract was also better than the remaining myrtle fruit parts. According to these results, myrtle seed is the structure within the fruit that has the strongest activity, probably due to the presence of galloyl derivatives [107]. Later on, these authors [108] studied the chemical composition and antioxidant activity of oil and methanolic extract of seeds of other variety of myrtle, *M. communis* var. *baetica*. The total phenol (25.25 mg/g), tannins (20.33 mg/g), flavonoids (0.75 mg/g), and proanthocyanidins (0.75 mg/g) were determined in the methanolic extracts. The capacity of this extract for scavenging DPPH free radicals (IC_50_ = 0.01 mg/mL), preventing the bleaching of β-carotene (IC_50_ = 0.07 mg/mL), chelating activity (3 mg/mL), and reducing power (0.01 mg/mL) were determined by the authors. The IC_50_ values found in this extract [108] were not significantly different to those reported for the methanolic extract of *M. communis* var. *italica* [107].

Babou et al. [109] also studied the chemical composition, antioxidant activity (ability for scavenging DPPH, superoxide, and nitric oxide free radicals) and inhibition of acetylcholinesterase of different parts of myrtle fruits and leaves. Simultaneously, they studied the influence of extraction processes (sonication followed by maceration with methanol:water 1:1 and decoction using water) and maturation stage (collection of plant material in September and December) on the chemical composition. The phenolic composition is depicted in Table 2. The concentrations of polyphenols in the extracts were dependent on both plant organ and extraction procedure. Hydroxybenzoic acids predominated in both seed extracts, whereas anthocyanins were at higher concentration in the pericarps (December), independent on the type of extract. Leaf aqueous extract from samples of December had higher amounts of flavonol glycosides and flavonol aglycones than the remaining samples, however, these groups of compounds were higher in methanolic/aqueous extract of leaves collected in September and seeds collected in December, respectively. Aqueous extracts extracted more amounts of phenols than the methanolic/aqueous extract. The capacity for scavenging DPPH free radicals, there was no significant difference between leaves-September (IC_50_ = 8.29–8.45 μg/mL) and leaves-December (IC_50_ = 9.44–9.51 μg/mL), berries-September (IC_50_ = 8.42 μg/mL) and seeds-December (IC_50_ = 3.89–6.50 μg/mL) samples. Significant differences in the capacity for scavenging the superoxide anion radical were not observed by the authors in both extraction procedures (except for pericarps-December extracts). The most active extracts were those of leaves-September (IC_50_ = 29.70–31.69 μg/mL) and leaves-December (IC_50_ = 33.70–34.69 μg/mL), berries-September (IC_50_ = 28.55–31.49 μg/mL) and seeds-December (IC_50_ = 24.19–28.32 μg/mL). Aqueous extracts of leaves-September (IC_50_ = 17.81 μg/mL), leaves-December (13.69 μg/mL), berries-September (22.16 μg/mL), and seeds-December (20.00 μg/mL) had the highest capacity for scavenging nitric oxide radicals [109]. The contribution of each phenolic compound in the antiradicalar activity was not evaluated by the authors but a statistical treatment allowed them to observe that the compounds contributing most for the antioxidant activity were the hydroxybenzoic acids (gallic and ellagic acids) and the flavonols (quercetin, quercetin-3-*O*-galactoside, quercetin-3-*O*-rutinoside, myricetin-3-*O*-rhamnoside, myricetin, and kaempferol). Although the highest amounts of phenols in both extracts of seeds, the anticholinesterase activity was weak [109].

Generally, maceration is the most common procedure for extracting phenols from myrtle berries, with some exception as reported above, in which the authors assayed other methods to compare the efficiency of phenol extraction, such as sonication or decoction [109]. Sonication or ultrasound-assisted extraction was also assayed by Pereira et al. [112] and supercritical fluid extraction [111] for extracting phenols and for evaluating the antioxidant activity of such extracts obtained from leaves and berries of Portuguese *M. communis* L. Flavonoids from the family of quercetin and myricetin were present in the myrtle leaf extracts obtained by ultrasound-assisted extraction, and anthocyanins, hydrolysable tannins, and quinic acid (Table 2) were the constituents found in berries obtained by the same extraction method [112]. The antioxidant activity determined through the methods of ABTS and ORAC (oxygen radical antioxidant capacity) that measures the ability of samples for scavenging peroxyl free radicals, correlated with the phenol content, although the authors had not determined the contribution of individual phenol for the ability of quenching the free radicals. The samples were analysed by HPLC–DAD–ESI–MS/MS. The leaf extracts had higher antioxidant activity (358 μmol Trolox/g and 624 μmol Trolox/g) than berries extracts (179 μmol Trolox/g and 366 μmol Trolox/g) for ABTS and ORAC methods, respectively, although the anthocyanin content is quite high. Such finding may indicate that the best activity comes from the compounds belonging to the flavonols and not from the anthocyanins [112].

Pereira et al. [111] evaluated two extraction procedures of secondary metabolites from Portuguese leaves and berries of *M. communis* (liquid phase extraction and supercritical fluid extraction) on the composition and concentration of phenols (HPLC-DAD-MS/MS methods), and antiradicalar activity (ability for scavenging ABTS and peroxyl free radicals), during three years. In the liquid phase extraction, the extracts were obtained from the water dearomatized by hydrodistillation that was extracted with diisopropyl ether, whereas in the supercritical fluid extraction, the extracts were obtained at 23 MPa, 45 °C and a CO_2_ flow of 0.3 kg/h using ethanol as cosolvent with a flow rate of 0.09 kg/h. The compounds found by the authors are listed in Table 2, flavonoids and anthocyanins were found in those extracts obtained by supercritical fluid extraction, whereas phenolic acids were only observed in the extracts obtained by liquid phase extraction. Extracts obtained by supercritical fluid extraction exhibited higher concentration of phenols and higher antioxidant activity, correlating well with the concentration of flavonol glycosides, the myricetin-*O*-glycosides [111]. In addition, leaf extracts were more active as antioxidants than berries in line with that already observed by [112] when used ultrasound-assisted extraction. The antiradicalar activities found by the authors were: ABTS (Leaves): 55–130 μmol Trolox/g; ABTS (Berries): 25–80 μmol Trolox/g; ORAC (Leaves): 530–759 μmol Trolox/g; ORAC (Berries): 130–250 μmol Trolox/g. With the exception of ORAC (Leaves), the activities found by Pereira et al. [111] for the extracts obtained by supercritical fluid extraction were lower than those obtained by ultrasound-assisted extraction. Such results demonstrated the importance of the extraction method on the antioxidant activity. The differences found by the authors [111] in the phenol content and antioxidant activity of the samples collected at different years were attributed to climatic factors since polyphenol content is affected by temperature [111].

The best antioxidant activity (DPPH radical scavenging capacity assay, the reducing antioxidant power assay and β-carotene linoleic acid assay) of leaf extracts had been already reported [113] for the different extract solvents (methanolic, ethanolic, and aqueous) of Moroccan myrtle. The amounts of total phenols were also higher in leaf extracts, independent on the extract, than berry extracts. The total phenol content of myrtle extracts ranged between 9.0 and 35.6 mg gallic acid equivalent/g extract. In leaf extracts, the overall antioxidant strength was in the order methanol > water > ethanol, whereas in berry extracts the order was methanol > ethanol > water. A positive correlation between the phenolic content with the antioxidant activity was observed by the authors: DPPH assay showed the highest correlation (*r*^2^ = 0.949), followed by the reducing power assay (*r*^2^ = 0.914) and the lowest for the β-carotene linoleic acid assay (*r*^2^ = 0.722).

Later on, Amensour et al. [114] evaluated the amounts of total phenols and flavonoids of extracts of Moroccan leaves and berries of *M. communis* extracted with methanol, ethanol, ethyl acetate, and water. At the same time, they determined the antiradicalar capacity of all of these extracts using the ABTS method. Once again, the authors found that leaf extracts, independent on the extraction solvent, had higher activity that the remaining extracts. This higher activity is also coincident with the highest concentration of flavonoids and total phenols. In leaf and berry extracts, the overall antioxidant strengths were in the order methanol > water > ethanol > ethyl acetate, which were coincident with the order of flavonoids’ concentration. In leaf and berry extracts, the order of total phenols was: water > methanol > ethanol > ethyl acetate. In addition, the authors observed higher positive correlation between capacity for scavenging the ABTS free radicals and total phenols (*r*^2^ = 0.9452) than capacity for scavenging the ABTS free radicals and total flavonoids (*r*^2^ = 0.5978), whereby the authors suggested that apart from flavonoids, there might be other phenolic compounds such as phenolic acids, tannic acid, and others responsible for the antioxidant activity.

Beyond the antioxidant activity of berry extracts, Serio et al. [115] also evaluated the anti-listerial activity. The hydroalcoholic extracts of red berries of *M. communis* exhibited antilisterial activity (two type strains and four isolates) and antioxidant activity (capacity for scavenging the ABTS free radicals) [115]. The authors used Central Composite Design (CCD) for studying the combined effect of sub-lethal concentrations of myrtle extract, NaCl (0–2.0 g/100 mL) and pH (5–7) on the growth of the six *Listeria monocytogenes* strains. The highest myrtle extract concentrations (0.117–0.195 mL/100 mL) combined with the lowest pH values (5.0–6.0) inhibited the growth of *L. monocytogenes*. This extract also possessed antioxidant activity that was stable during 70 days of storage in refrigerated conditions. According to the authors [115], such will permit to use this type of extracts with a certain quality assurance. The polyphenol content of the same extract was 5315.20 mg gallic acid equivalent/kg, and malvidin-3-*O*-glucoside was the most abundant anthocyanin in the same extract.

#### 2.3. Anti-Inflammatory Activity of Berry Extracts

The anti-inflammatory activity of four species (*Myrtus communis*, *Smilax aspera*, *Lavandula stoechas*, and *Calamintha nepeta*) was evaluated by Amira et al. [116]. At the same time, the authors sought a possible correlation of anti-inflammatory activity with the antioxidant activity. The anti-inflammatory activity was done through the method of the carrageenan-induced paw oedema and the antioxidant activity through the following methods (DPPH, ABTS, galvinoxyl, superoxide and peroxynitrite scavenging activities, reducing power, and human plasma lipid peroxidation). Myrtle extract had the highest inhibitory activity in the paw oedema induced by carrageenan (60% at 3 h), in contrast to lavender that had the lowest inhibitory property (38%). Myrtle extract was the best among the extracts studied for scavenging the DPPH (163 μg Trolox equivalent/mg), ABTS (726 μg Trolox equivalent/mg) free radicals as well as reducing power (1351 μg ascorbic acid equivalent/mg); nevertheless *C. nepeta* extract was the best for scavenging galvinoxyl and superoxide radicals and peroxynitrite anion. *M. communis* extract was even unable to scavenge this anion, at least at the higher concentration tested (100 μg/mL) [116]. The inhibition of human plasma lipid peroxidation, assayed through the thiobarbituric acid reactive substance method, was higher in *C. nepeta* and *L. stoechas* extracts (>80%), while the inhibition percentage observed for myrtle extract was <25%. Reactive oxygen species, such as superoxide anion, peroxynitrite anion, and hydroxyl radicals, produced by neutrophils, have a role in the inflammatory processes, therefore, compounds able to scavenge or inhibit the production of these radicals may have a positive role in the inflammation. Myrtle extract had lower concentration of total phenols (117 μg quercetin equivalent/mg) than *L. stoechas* or *C. nepeta*, nevertheless exhibited high ability to reduce the FRAP reagent or scavenge the DPPH and ABTS free radicals, and the best anti-inflammatory activity, whereby the activities found cannot be attributed to the total amount of phenols, but to the type of phenolics or other compounds not quantified [116]. In addition, the best anti-inflammatory activity of myrtle observed by the authors cannot be attributed to its ability for scavenging superoxide anion and peroxynitrite anion, since the activities were low or even null.

The antidiarrheal effects of myrtle berries seeds aqueous extracts from Tunisia and their antioxidant activity were determined by Jabri et al. [32] in adult male Wistar rats. According to the authors, castor oil induces intestinal hypersecretion and diarrhoea, which is accompanied by an oxidative stress. Myrtle berries seeds aqueous extracts were able to reduce the intestinal fluid accumulation protecting against diarrhoea, and decreasing the oxidative stress, particularly reducing hydrogen peroxide, and free iron levels in a dose-dependent manner. Acute castor oil also increases lipoperoxidation with higher accumulation of malondialdehyde and decreases the thiol groups in intestinal mucosa, which is reversed by administering myrtle berries seeds aqueous extracts [32]. Eighteen compounds belonging to three major groups (hydroxybenzoic acid derivatives, anthocyanins derivatives, and flavonols derivatives) were identified in the extracts (Table 2).

The gastroesophageal reflux disease occurs because there is a lower esophageal sphincter dysfunction, decreased esophageal clearance capacity, esophageal mucosal barrier dysfunction, esophageal visceral hypersensitivity, and increased gastric acid secretion [117]. The chronicity of this disease leads to erosions, stenosis, ulcer, or metaplastic epithelium of lower esophagus [117]. Inflammatory cytokines (interleukin-6, IL-6 and interleukin-8, IL-8), leukocytes, and oxidative stress seem to have an important role in the development of the gastroesophageal reflux disease [118]. For this reason, Jabri et al. [117] determined the protective effect of the myrtle berry seed aqueous extract against gastroesophageal reflux, not only evaluating its capacity for scavenging *in vitro* ABTS free radicals and hydrogen peroxide, but also evaluating the free radical scavenging activities of plasma using the DPPH radical method, the capacity for preventing esophageal lipid peroxidation measured through the malondialdehyde (MDA) determination, as well as the influence of the extract on the nonenzymatic antioxidant levels and antioxidant enzyme activities (superoxide dismutase, catalase, and glutathione peroxidase). According to the authors [117], the effective concentrations 50 (EC_50_) for ABTS and hydrogen peroxide scavenging activities were 184.34 and 380.96 μg/mL, respectively, higher than the control gallic acid (73.34 and 324.31 μg/mL, respectively), therefore poorer than the control. The oxidative stress in the esophageal tissue was significantly decreased (lower MDA accumulation), and the plasma scavenging activities, the esophageal nonenzymatic antioxidant levels and the antioxidant enzyme activities increased in a dose-dependent manner when animals (male Wistar rats) were treated with the extract or the controls (gallic acid and famotidine). In addition, the authors [117] also observed that the extract restored the pH that decreased in the presence of gastroesophageal reflux disease. The authors attributed these beneficial properties to the high amounts of total polyphenols (147.56 mg gallic acid equivalent/g) and total anthocyanins (5.01 cyanidin 3-glucoside equivalent/g) in the aqueous extract of myrtle berry seeds, although no correlation had been made by them [117]. In ulcerative colitis there is also an oxidative stress with the production of reactive oxygen species. Jabri et al. [73], studying the effect of aqueous extracts of berry seeds of myrtle against acetic acid-induced colonic lesions in rats, found that they decreased the formation of malondialdehyde, therefore decreased the colonic lipoperoxidation, and increased the nonenzymatic antioxidant levels, thiol groups, and glutathione, and the activity of superoxide dismutase, catalase, and glutathione peroxidase. The aqueous extract was predominantly constituted by phenols (Table 2).

#### 2.4. Antioxidant Activity of Berry Foods

The evaluation of antioxidant activities of myrtle berries was predominantly *in vitro*, as reported above, with some very few exceptions, as those described above in which *ex vivo* and *in vivo* assays were used. The application of myrtle berry extracts in foods is also limited, although some works could be found and reported below.

Beyond the application of myrtle berries in liqueurs, they can also be used for making jam. Rosa et al. [119] evaluated the antioxidant activity of methanol extracts of myrtle berries jam and compared their results with other extracts obtained from prickly pear fruit jam and cream, and orange and mandarin-orange marmalades. The chemical profile of methanol extracts was characterized by ^1^H-NMR (proton-nuclear magnetic resonance) spectroscopy. The antioxidant activity was followed through the determination of capacity of samples for preventing lipid peroxidation using liposomes as lipid substrate and measuring the inhibition of malondialdehyde production and the capacity for scavenging reactive oxygen species using the 2′,7′-dichlorofluorescein diacetate (DCFH-DA) in Caco-2 cells. The results showed that the extract of myrtle jam exhibited antioxidant activity, nevertheless the authors considered two possible factors responsible for such property: amount of phenols (206.33 gallic acid equivalent/100 g) found in the sample and the products from nonenzymatic browning reactions resulting from the jam production [119]. Extracts from prickly pear cream and myrtle berries jam preserved liposomes from oxidation, and extracts from prickly pear cream and citrus marmalades significantly reduced the reactive oxygen species generation in Caco-2 cell culture. Using the ^1^H-NMR, the authors did not identify phenolic compounds in the methanolic extract of myrtle berries jam [119].

In the ice cream formulation, sometimes prebiotics, such as dietary fibers or oligosaccharides, were added. The utilization of fruits as prebiotics in ice cream formulation is scarce. Öztürk et al. [120] used dark blue and white myrtle berries along with the probiotic *Lactobacillus casei* (*L. casei*) 431 in the ice cream formulation. The aim of the work was to study the performance of *L. casei* strain in ice cream during frozen storage in association with dark blue and white myrtle berries. The sensory acceptability of the new formulation was also evaluated. The results showed that *L. casei* 431 kept viable throughout the storage period and increased with the freezing process, and the addition of myrtle fruits lead to an increase of total phenols (5 and 8 mg gallic acid equivalent/g, in the presence of white and dark blue berries, respectively), although the antioxidant activity had not undergone any alteration during the same storage period. The addition of pulp fruits to the ice cream with *L. casei* 431 improved the antioxidant activity, showing a positive effect of fruits on the probiotic *L. casei* 431. Ice cream samples with *L. casei* 431 and dark blue berries of myrtle exhibited higher antioxidant activity (EC_50_ = 90.25 after 8 weeks of storage—85.48 mg/L, on day 1) than when white pulps were added (EC_50_ = 263 after 8 weeks of storage—323 mg/L, on day 1), which may be explained by the highest total phenol content found in the ice cream with *L. casei* 431 and dark blue berries (22.5–26.5 mg gallic acid equivalent/100 g). In ice-cream formulation with *L. casei* 431 and white berries, the amounts of total phenols ranged from 8 to 13.5 mg gallic acid equivalent/100 g. The sensory was improved with the addition of myrtle fruits, particularly white ones, because the formulation in which *L. casei* 431 and white myrtles were added, the acidic taste characteristic of a fermentation process, was eliminated. With these results the authors suggest that dark blue and white berries should be used together in new probiotic product formulations [120].

Curiel et al. [121] used a selected lactic acid bacterium (*Lactobacillus plantarum* C2, which was previously isolated from carrots, identified by partial sequencing of 16S rRNA) in myrtle berries with the objective to improve their antioxidant activity and, consequently, to enhance the functional properties of *M. communis*. The authors determined the antioxidant activity either *in vitro* (capacity for scavenging ABTS and DPPH free radicals and lipid peroxidation inhibitory activity) or *ex vivo* on murine fibroblasts Balb3T3 using the dichloro-dihydro-fluorescein diacetate (DCFH-DA) method, which measures the intracellular reactive oxygen species generation, after analysing the cytotoxicity of extracts through the MTT (3-(4,5-dimethyl-2-yl)-2,5-diphenyltetrazolium bromide) method. Myrtle berries with yeast extract (0.4%) and fermented with L. *plantarum* C2 had significantly higher antioxidant activity *in vitro* than the control constituted by acidified homogenate without bacterial inoculum and submitted to the same experimental conditions. The antiradicalar activity, measured through the DPPH method, increased by 30% and the capacity for inhibiting linoleic acid peroxidation increased twice when compared to the control. The phenols (gallic and ellagic acids), flavonoids (myricetin and quercetin), and anthocyanins’ contents also enhanced in the fermented samples, about 5–10 times higher than those found for the nonfermented samples (Table 2). The highest increase of gallic and ellagic acids can be attributed to tannase or tannin acyl hydrolase of *L. plantarum* that catalyzes the hydrolysis of ester bonds present in hydrolysable tannins and gallic acid esters [121]. Other esterases may also be responsible for the increase of the aglycones myricetin and quercetin in the fermented homogenates. The antioxidant activity of fermented homogenates was confirmed ex vivo. The results show that the antioxidant activity of myrtle berries can be improved through lactic acid fermentation [121].

### 3. *Myrtus communis*: Leaves

#### 3.1. Antioxidant Activity

The antioxidant activity of leaf extracts of myrtle has also been deeply studied as well as their phenolic profiles that are somehow different from those of berries (red and dark blue ones), at least in the absence of anthocyanins. This was already reported in the previous section [109,111,112,114].

The effect of various factors on the antioxidant activity of myrtle leaves has deeply studied. The chemical profile and biological properties of plants can be affected by climatic conditions, harvesting time, abiotic stress, genotype among other factors [123]. For this reason, the authors [123] studied the effect of different NaCl concentrations (control, 2, 4, and 6 dS/m) and three harvesting times in different seasons including spring, summer, and fall on the phenolic, flavonoid content, and antioxidant activity (DPPH radical scavenging activity, reducing power, and β-carotene/linoleic acid bleaching test) of myrtle extracts. The highest antioxidant activity was found in plants harvested in summer and spring and in high stress condition. In the DPPH test, the lowest IC_50_ values were obtained in 6 dS/m in summer (249.41 μg/mL), followed by spring (375.23 μg/mL), and fall (618.38 μg/mL). The chemical composition is described in Table 3. After the sum of the compounds identified in the myrtle extracts, it is possible to verify an increase of phenols plus flavonoids since spring (131.26 mg/100 g) up to the fall (260.87 mg/100 g). The contribution of phenols on the antioxidant activity was not determined by the authors, but strong correlation between phenol and flavonoid contents and the DPPH test, reducing power, and β-carotene/linoleic acid bleaching test was detected. Sacchetti et al. [124] after harvesting myrtle plants at different places of Sardinia also reported the different capacity of myrtle extracts to scavenge the DPPH free radicals.

The identification of the compounds that constituted the essential oils and methanolic extracts isolated from leaf, flower, and stems of Tunisian *M. communis* var. *italica* and the antioxidant activity (DPPH radical scavenging, β-carotene-linoleic acid bleaching, reducing power, and metal chelating activity assays) were determined by [125]. The amounts of total phenols, and condensed tannins and flavonoids were different according to the plant part from where they were extracted (Table 3). The analysis indicated that the main phenolic class was hydrolysable tannins (gallotannins) in leaf (8.90 mg/g) and flower (3.50 mg/g) while in the stem predominated flavonoid class (1.86 mg/g) due to the high presence of catechin (1.12 mg/g) (Table 3). In almost all antioxidant tests, leaf extracts had the best activity, presenting the lowest IC_50_ values, the exception was in DPPH method that flower extract had the lowest IC_50_ value. For DPPH assays, the IC_50_ values for leaf, stem and flower were 8 μg/mL, 90 μg/mL, and 3 μg/mL, respectively. For β-carotene-linoleic bleaching test, the IC_50_ values were: 70 μg/mL, 124 μg/mL, and 78 μg/mL, respectively. For chelating activity, the IC_50_ values were 5 μg/mL, 10 μg/mL, and 46 μg/mL, respectively, whereas for reducing power, such values were 10 μg/mL, 150 μg/mL, and 50 μg/mL, respectively. In comparison with essential oils, the methanolic extracts exhibited higher antioxidant activity, showing the importance of the presence of phenols in the samples [125].

#### 3.2. Comparison of Antioxidant Activity of Myrtle Leaves with Other Plant Species

In several works, the authors compare the antioxidant activities of extracts obtained from myrtle leaves with those obtained from other species and the results can be sometimes quite different, as described below.

The antioxidant activity of selected medicinal plants from the North-West of Morocco (*Origanum compactum* Benth., *Cistus crispus* L., *Centaurium erythraea* Rafin., *Myrtus communis* L., and *Arbutus unedo* L.) were tested for their anticancer and antioxidant activities. The antioxidant activity was evaluated using the reducing power activity and the capacity for scavenging the ABTS free radicals. The authors also evaluated the effect of extraction solvent on the activities (methanol, ethanol, and *n*-hexane) [60]. All extracts were able to scavenge the free radicals and have ferric-reducing power, nevertheless dependent on the plant and type of extracts. The methanol and *n*-hexane extracts of myrtle and the methanolic extract of *C. erythraea* showed important antioxidant capacity to scavenge ABTS free radicals (IC_50_ = 57.83, 48.42, 63.48 μg/mL, respectively), and to reduce ferric to ferrous ions (IC_50_ = 16.59, 23.8, 27.28 μg/mL, respectively), but their IC_50_ values were inferior than the positive controls used (Trolox and ascorbic acid), therefore, showed better antioxidant activity [60].

The antioxidant activity, measured through the capacity for scavenging the DPPH and nitric oxide (NO) free radicals, β-carotene-bleaching test and metal chelating power, of six plants (*M. communis*, *Eryngium maritimum*, *Pistacia lentiscus*, *Globularia alypum*, *Marrubium vulgare*, and *Scilla maritima*) was determined by [126]. The total phenols, total flavonoids, flavonols, proanthocyanidins, and total tannins were also evaluated. The authors observed that methanol extracts of *M. communis* (leaves) (285.73 mg gallic acid equivalent/g), *P. lentiscus* (leaves) (238.33 mg gallic acid equivalent/g), and *G. alypum* (flowers) (156.97 mg gallic acid equivalent/g) presented the highest amounts of total phenolic compounds while the concentrations of total flavonoids, flavonols, proanthocyanidins, and total tannins varied with plant species. In the DPPH assay, *P. lentiscus* (IC_50_ = 0.008 mg/mL) and *M. communis* (IC_50_ = 0.003 mg/mL) had the best activity and their inhibitions were similar. In the β-carotene assay, leaf and fruits extracts of *M. communis* and *P. lentiscus* leaves were the most potent with 63.60%, 47.61%, and 43.02%, respectively. Metal chelating activity assay showed that *E. maritimum* leaves and stems and *M. communis* leaves had the best chelating power, 49.78%, 32.32%, and 35.98%, respectively. These results indicate that *M. communis* extracts present good antioxidant activity, being even better than other plants from Algeria [126]. Myrtle extracts did not exhibit any anti-inflammatory activity when determined through the inhibition of cyclooxygenase-1-inhibition, nevertheless, it was the best extract for inhibiting acetylcholinesterase activity along with the *P. lentiscus* with IC_50_ values of 0.03 and 0.01 mg/mL, respectively [126].

The best antioxidant activity of myrtle extracts amongst two sets of sixteen and four plant extracts was also previously reported by [127,128], regardless the extraction solvent (hexane or methanol). β-Carotene-bleaching test was used for determining the antioxidant activity and the results were presented as antioxidant activity coefficients. In the set of sixteen samples, the antioxidant activity coefficients for hexane and methanol extracts obtained from fresh leaves were 641 and 260, respectively, whereas for dried leaves, the values were 12.2 and 51.5, respectively [127]. In both fresh and dried material, myrtle extracts were the most active, nevertheless in the second set of extracts [128], the highest antioxidant activity coefficient was observed for fresh extract of *Myrtus communis* (AAC = 635), whereas in dried material, *Thymus vulgaris* was the most active (antioxidant activity coefficient = 34). The good antioxidant activity of hexane extracts can be partly or wholy assigned to the presence of nonpolar phenolic compounds such as tocopherols. Demo et al. [129] detected and quantified α-tocopherol in hexane extracts of myrtle leaves (2.144%).

Mothana et al. [35] studied the antimicrobial, anticancer, and antioxidant activity of 32 Yemeni plants. Concerning the antixoxidant activity and within this set of samples, only six had high DPPH free radical scavenging activity: methanolic extracts of *Achillea biebersteinii*, *Chrozophora oblongifolia*, *Myrtus communis*, *Oxalis corniculata*, *Phragmanthera regularis,* and *Tecoma stans* at 50 μg/mL. In contrast, Özcan et al. [130] did not find similar results for Turkish myrtle, although the methods (ABTS radical scavenging activity and capacity for oxidizing ferrous ion to ferric ion by various types of peroxides within the plasma) and the species used were different (anise, bitter fennel fruits and flowers, basil, laurel, oregano, and pickling herb). The peroxide value and the Trolox equivalent of methanolic extracts were 0.6866 μmol H_2_O_2_ and 0.3189 Trolox equivalent/g, respectively, although presenting higher amounts of total phenols (9.9761 mg gallic acid equivalent/g) than the other species (1.3175–10.5832 mg gallic acid equivalent/g). Only oregano extracts presented higher concentration of total phenols than myrtle leaves. The authors [130] also evaluated the antioxidant activity of the essential oils. These ones had better capacity for reducing ferrous ions, since they showed higher peroxide values, than the methanolic extracts, nevertheless poorer capacity for scavenging the ABTS free radicals.

Gião et al. [131] determined the antiradical activity (ABTS free radicals) of aqueous extracts of 32 plants from Portugal. Two types of extraction were used: boiling water added to the sample and left during 5 min at rom temperature (infusion), and water added to the sample and the mixture heated until boiling, which was maintained for 5 min. The authors detected that the antiradicalar activity was dependent on the species as well as of the method of extraction. The highest antioxidant activity was observed for avocado (0.157 mg equivalent ascorbic acid/g), followed by agrimony (0.067 g equivalent ascorbic acid/g), eucalyptus (0.149 g equivalent ascorbic acid/g), yarrow (0.118 g equivalent ascorbic acid/g), myrtle (0.141 g equivalent ascorbic acid/g), thyme (0.142 g equivalent ascorbic acid/g), and heath (0.065 g equivalent ascorbic acid/g). In addition, powder infusion was the best method for obtaining the most active extracts which also possessed the highest amounts of phenols. The authors found a positive correlation between the phenol content and the antiradicalr activity [131]. Later on, Gião et al. [132] studied the effect of different stages of processing (fresh, frozen, dehumidified/packed in two consecutive years, and storage after dehumidification under controlled relative humidity, maintained for one year in a dark room) on the antiradicalar activity of ten species (agrimony (*Agrimonia eupatoria*), eucalyptus (*Eucalyptus globulus*), walnut-tree (*Juglans regia*), myrtle (*Myrtus communis*), raspberry (*Rubus idaeus*), sage (*Salvia* sp.), savory (*Satureja montana*), sweet-amber (*Hypericum androsaemum*), thyme (*Thymus vulgaris*), and yarrow (*Achillea millefolium*)). The samples used were infusions and the antiradicalar method used was based on the abilty for scavenging ABTS free radicals. According to the authors [132], antioxidant activity and total phenolic content decreased by ca. 30–80%, between fresh and frozen forms, whereas from the frozen stage to the packaged form the variations observed were not statistically significant. The highest difference was observed for myrtle, which means that this species is sensitive to the technological processing and, therefore, for preserving their properties during processing conditions after harvest and throughout storage, other techniques must be thought and assayed in the near future.

Gonçalves et al. [133] compared the antioxidant activity of ten plant species from Portugal, including *M. communis*, measured through diverse methods (DPPH and hydroxyl radical scavenging activity, reducing and chelatin power, and inhibition of lipid peroxidation in mouse brain homogenates using thiobarbituric acid reactive substances). The extracts obtained were aqueous obtained by maceration at room temperature, for 2 h, or extraction in hot water (90 °C), for 5 min. *Pistacia lentiscus* L. and *M. communis* in cold and hot aqueous extracts were the most effective for scavenging the DPPH free radicals (377.30 and 319.81 mmol Trolox equivalent/g and 230.36 and 246.51 mmol Trolox equivalent/g, respectively). The same extracts were also the best ones for chelating iron ions without significant differences between the hot and cold extracts. However, the capacity for scavenging hydroxyl radicals was better in hot extracts, nevertheless never exceeding 50%, even at higher extract concentration (1.6 mg/mL extract). With the exception of this activity, the authors found a positive correlation between the total phenolic content and the antioxidant activity [133]. Concerning the capacity for preventing lipid peroxidation, all samples had activity, although those of *Centaurea erythraea*, *Paronychia argentea*, and *Ruscus aculeatus* were significantly less active than the other aqueous extracts.

#### 3.3. Effect of Extraction Method and Extraction Solvent on the Antioxidant Activity of Myrtle Leaves

The antiradicalar activity of leaf extracts measured through the capacity for scavenging DPPH as well as the total antioxidant power were evaluated by Belmimoun et al. [134]. The authors used diverse methods and extraction solvents: decoction, maceration with ethanol, and extraction with solvents of increasing polarity by Soxhlet (dichloromethane and methanol). The IC_50_ value for the aqueous extract was 29 μg/mL, whereas the total antioxidant power was 68.05 mg/g, better when compared to the essential oils obtained by hydrodistillation (615 μg/mL and 36 mg/g, respectively), explained by the absence of phenol compounds in the essential oils [134].

Romani et al. [135] also evaluated the influence of different solvents on the antioxidant activity of leaf myrtle extracts obtained by liquid–liquid extraction. The authors also evaluated the role of pure compounds and group of compounds found in the myrtle extracts on the antioxidant activity found in the work. As expected, different solvents extracted diverse phenolic compounds. Hydroalcoholic extracts had galloylglucosides, ellagitannins, galloyl-quinic acids, and flavonol glycosides; whereas ethyl acetate extract and aqueous residues after liquid–liquid extraction were enriched in flavonol glycosides and hydrolysable tannins (galloyl-glucosides, ellagitannins, and galloyl-quinic acids), respectively (Table 3). The antioxidant activity of extracts was determined evaluating the capacity of extracts to prevent the formation of MDA and conjugated dienes after exposing human LDL to copper ions. Hydroalcoholic extract was mainly constituted by galloyl-glucosides and ellagitannins and was the most active in inhibiting LDL oxidation (IC_50_ = 0.36 μM) followed by the aqueous residue after liquid–liquid extraction (IC_50_ = 2.88 μM), also mainly constituted by galloyl-glucosides and ellagitannins, and ethyl acetate extract (IC_50_ = 2.27 μM), mainly constituted by myricetin glucosides and galloylquinic acids. The capacity for preventing MDA accumulation was also determined using pure compounds such as gallic acid, 3,5-di-*O*-galloylquinic acid, myricitrin, and rutin, and the IC_50_ values found were 20, 2.2,7.8, and 3.7 μM, respectively, showing that 3,5-di-*O*-galloylquinic acid was the most active. In addition, the authors [135] also determined the IC_50_ values considering the total polyphenol concentration and the concentration of the single compound present in each extract and it was possible to find that the copresence of different polyphenols increased the antioxidant activity. Two examples are the aqueous residue in which was practically constituted by galloyl-glucosides and had a IC_50_ value close to that of 3,5-di-*O*-galloylquinic acid, and the other example is that of the ethyl acetate extract that had a ratio between galloyl derivatives and flavonols of about 1:1, and the IC_50_ value is very similar to that of 3,5-di-*O*-galloylquinic acid. This last result indicates that flavonols do not play an important role in the antioxidant activity when they are mixed with hydrolysable tannins, but a ratio of 9:1, such as observed in the hydroalcoholic extract, the IC_50_ decreased drastically, that is, the activity increased. The authors [135] concluded that the antioxidant activity was dependent on the ratio between the sum of galloylglucosides, ellagitannins, and flavonols and also of the ratio between these galloyl derivatives vs. galloyl-quinic acids.

The effect of different solvents (water, hexane, chloroform, ethyl acetate, methanol, and a total flavonoids oligomer fraction) and essential oils on the capacity for scavenging DPPH free radicals revealed that the aqueous extract was the most active (IC_50_ = 1.9 μg/mL), even better than the total flavonoids oligomer fraction (IC_50_ = 3 μg/mL). Chloroform, hexane extracts, and essential oils were significantly less active than those extracts [66]. Later on, Hayder et al. [64] evaluated the capacity of myricetin-3-*O*-galactoside and myricetin-3-*O*-rhamnoside (flavonoids), isolated from the leaves of *Myrtus communis*, to inhibit xanthine oxidase activity, lipid peroxidation, and to scavenge the free radical DPPH. Both flavonoids were able to scavenge the free radicals with IC_50_ values of 1.4 μg/mL and 3.1 μg/mL for myricetin-3-*O*-rhamnoside and myricetin-3-*O*-galactoside, respectively, comparable than to that of the positive control (vitamin E) (IC_50_ = 3 μg/mL). Both flavonoids were able to inhibit xanthine oxidase (in the catalysis process there is production of superoxide radical anions). At 100 μg/mL, myricetin-3-*O*-rhamnoside and myricetin-3-*O*-galactoside showed percentages of inhibitory activities of 59% and 57%, respectively. However, when higher concentrations of myricetin-3-*O*-rhamnoside (300 μg/mL) and myricetin-3-*O*-galactoside (200 and 300 μg/mL) were used, there was an increase of xanthine oxidase activity, that is, a pro-oxidant activity. The inhibition of the malondialdehyde formation by K562 (human chronic myelogenous leukemia) cell line, induced by hydrogen peroxide, was also assayed, and the authors [64] found that the IC_50_ values for myricetin-3-*O*-galactoside and myricetin-3-*O*-rhamnoside were 160 and 220 μg/mL, respectively, measured through the thiobarbituric acid test. These concentrations did not involve a decrease of cell viability.

The evaluation of antioxidant activity of pure compounds isolated from myrtle leaves was also reported previously [65]. In this case, the compound studied was 3,5-*O*-digalloylquinic acid and the antioxidant activity was determined by its ability for inhibiting lipid peroxidation induced by hydrogen peroxide in the K562 cell line. The pure molecule displayed an important malondialdehyde formation inhibition percentage (82.2%) and low IC_50_ value = 180 μg/mL. This concentration did not induce a decrease of cell viability whereby the decrease of malondialdehyde amounts can only be attributed to the real antioxidant activity of 3,5-*O*-digalloylquinic acid [65] as reported for myricetin-3-*O*-galactoside and myricetin-3-*O*-rhamnoside [64].

Tumen et al. [136] studied the effect of dichloromethane, acetone, ethyl acetate, and methanol extracts of myrtle leaf and berries on the ability for scavenging DPPH and *N*,*N*-dimethyl-*p*-phenylenediamine (DMPD) radicals, reducing power, and chelating activity. The authors reported that the polar extracts (acetone, ethyl acetate, and methanol extracts) exerted strong scavenging effect against DPPH and DMPD as well as good reducing power. However, the dichloromethane extract of the berries possessed the best metal chelation ability. Berry extracts were better antioxidants than leaf extracts, maybe to the highest concentrations of phenols in those extracts, according to the Tumen et al. [136].

Yoshimura et al. [137], using different solvents in the extraction of phenols from leaves of myrle from Japan, isolated, identified, and determined the capacity of every single compound for scavenging the DPPH free radicals. Among the compounds evaluated, the hydrolyzable tannins oenothein B, eugeniflorin D_2_, tellimagrandin I, and tellimagrandin II exhibited the best activity (IC_50_ = 6.12, 4.56, 8.00, and 7.62 μM, respectively).

Previously, Dairi et al. [138] studied the scavenging capacity of the ABTS and peroxyl radicals by Algerian myrtle extracts obtained by microwave assisted extraction and maceration. In addition, the authors also evaluated the antioxidant activity of leaf extracts in lipid system models oxidized *in vitro*: human LDL Cu^2+^-oxidation and AAPH-induced l-*α* phosphatidylcholine aqueous dispersion oxidation. The results showed that there were not differences in the amounts of phenols obtained by both methods and they presented strong ability for scavenging the ABTS free radicals, even better than BHA and α-tocopherol. The same extracts also exhibited higher capacity for scavenging the peroxyl free radicals than BHA but less effective activity than the references caffeic acid and myricetin 3-*O*-rhamnoside. In the lipid system (Cu^2^^+^-induced LDL system), both myrtle extracts, as well as myricetin 3-*O*-rhamnoside, were able to inhibit the production of conjugated dienes in a dose-dependent manner and to prolong the lag phase. When the AAPH-induced l-*α* phosphatidylcholine aqueous dispersion was used, both myrtle extracts were effective to prevent lipid oxidation, but less than myricetin 3-*O*-rhamnoside. The possible synergic effect of myrtle extracts, caffeic acid, and myricetin 3-*O*-rhamnoside on α-tocopherol-enriched phospholipid aqueous dispersions was also evaluated by the authors and they observed that no synergic or additive effect was observed between *α*-tocopherol and myrtle extracts or caffeic acid, but myricetin 3-*O*-rhamnoside had an additive effect. According to the authors [138], myrtle extracts, in which myricetin 3-*O*-rhamnoside can be found, can improve the antioxidant activity of complex lipid systems, stabilizing them [138].

The absence of significant differences in the amounts of total phenols in the extracts obtained by microwave-assisted extraction and maceration, reported by Dairi et al. [138], such was also verified by Dahmoune et al. [139] when compared the total phenol amounts of leaf myrtle extracts obtained by microwave-assisted extraction (162.49 mg gallic acid equivalent/g), ultrasound-assisted extraction (147.77 mg gallic acid equivalent/g), and maceration (128.00 mg gallic acid equivalent/g). However, the amounts of total flavonoids and tannins observed in the extracts were different depending on the extraction procedures. Microwave-assisted extraction was able to extract more flavonoids (5.02 mg quercetin equivalent/g) and tannins (32.65 mg quercetin equivalent/g) than ultrasound-assisted extraction (3.88 mg quercetin equivalent/g and 23.32 mg/g, respectively) and maceration (4.15 mg quercetin equivalent/g and 17.15 mg/g, respectively). The capacity for scavenging ABTS (IC_50_ = 38.20 mg gallic acid equivalent/mL), DPPH (IC_50_ = 16.80 mg gallic acid equivalent/mL), and peroxyl radicals (757.77 μmol Trolox equivalent/g) was also more effective when myrtle extracts were obtained by microwave-assisted extraction, than by other extraction processes. However, optimal microwave-assisted extraction conditions were needed in order to achieve these values of phenols and antioxidant activity. Such conditions were 42% ethanol concentration, 500 W microwave power, 62 s irradiation time, and 32 mL/g solvent to material ratio. Microwave-assisted extraction of myrtle leaf allowed shortening the extraction time about 14 and 15 times when compared to the ultrasound-assisted extraction and maceration, respectively [139].

According to Pereira et al. [140], supercritical fluid extraction does not present disadvantages for extracting natural products, on the contrary, it can present the advantage to make selective extractions by varying pressure and temperature. Taking into account these premises, the authors used the response surface methodology to optimize the supercritical carbon dioxide fluid extraction conditions to obtain myrtle leaf extracts. The optimal conditions obtained were: 23 MPa, 45 °C, and CO_2_ flow rate of 0.3 kg/h agreeing to those predicted by the response surface methodology model. The capacity for scavenging ABTS was inferior when compared to the ethanolic extracts of the same plant. Keeping those parameters constant, the authors [140] used ethanol as cosolvent and at different percentages to know if the increase of polarity of solvent could improve the antioxidant activity. The authors observed that the increase of ethanol content increased the antioxidant activity that could be by around 4 and 5 times for scavenging ABTS and peroxyl radicals, respectively, when the flow rate of ethanol was 0.09 kg/h.

As leaves and branches of myrtle are frequently consumed as an infusion and decoction, Messaoud et al. [141] studied the chemical composition, volatiles and phenols, and the antibacterial and antioxidant activities of leaf infusions prepared during three different times (5, 10, and 15 min). The phenolic compounds and their amounts found during those periods are depicted in Table 3. Phenolic acids and flavonol glycosides were the major group of infusions (7.64 to 14.28 μmol/g and 7.05 to 12.11 μmol/g, respectively), which variations depended on the time of heating (Table 3). Longer heating periods (15 min), higher was the amounts of phenols found in the myrtle infusion. The antioxidant activity was measured using four in vitro methods: DPPH (2,2-diphenyl-1-picrylhydrazyl) method, *β*-carotene bleaching test, chelating effect on ferrous ions, and ferric reducing power method. The results showed that the heating time influenced the antioxidant activity of myrtle infusions, independent on the method used. For DPPH method, the IC_50_ values found were: 356.14, 283.71, and 282.53 μg/mL in infusion samples after 5, 10, and 15 min of heating, respectively. For *β*-carotene bleaching test, the IC_50_ values found were: 247.91, 138.43, and 84.88 μg/mL in infusion samples after 5, 10, and 15 min of heating, respectively. For chelating effect on ferrous ions, the IC_50_ values found were: 223.56, 215.86, and 206.44 μg/mL in infusion samples after 5, 10, and 15 min of heating, respectively. For the ferric reducing power method, the values found were: 31.26, 35.81, and 38.93 mmol Fe^2+^/mL in infusion samples after 5, 10, and 15 min of heating, respectively. As observed for phenols, the antioxidant capacity of infusions increases as heating time also increases. Such results suggest that phenolic compounds are responsible for the activities found. In fact, the authors [141] found a linear correlation between the phenols content and DPPH radical scavenging activity (*r*^2^ = 0.709), *β*-carotene bleaching test (*r*^2^ = 0.831), and ferric reducing power method (*r*^2^ = 0.858).

#### 3.4. Acylphloroglucinols on the Antioxidant Activity of Myrtle Leaves

Generally, the antioxidant leaf extracts of myrtle are assigned to phenols, particularly hydrolizable tannins and flavonoids, nevertheless the acylphloroglucinols semimyrtucommulone and myrtucommulone A are also described as antioxidants. Rosa et al. [142] reported that these acylphloroglucinols had antioxidant activity because they were able to prevent the thermal (140 °C), solvent-free oxidation of cholesterol. Myrtucommulone A at 5 nmol and 10 nmol exerted a complete inhibition of cholesterol degradation after 1 h or 2 h, showing a 90% protection at 2.5 nmol and 50% protection at 5 nmol at 1 and 2 h, respectively. Semimyrtucommulone was less active than myrtucommulone A. A complete inhibition of the oxidative process of cholesterol was observed from 5 nmol at 1 h and 20 nmol at 2 h, showing a 70% protection at 10 nmol at 2 h [142]. The lipid peroxidation was determined having LDL as lipid substrate and the oxidation was induced by Cu^2+^. The results showed that both acylphloroglucinols preserved LDL from oxidative damage. In addition, a protective effect on the reduction of polyunsaturated fatty acids and cholesterol was also observed by inhibiting the increase of their oxidative products (conjugated dienes fatty acids hydroperoxides, 7β-hydroxycholesterol, and 7-ketocholesterol). According to these results, semimyrtucommulone and myrtucommulone A can be seen as dietary antioxidants with antiatherogenicity.

From the myrtle leaves, it was possible to isolate and identify myrtucommuacetalone, myrtucommulone M, myricetin, isousnic acid, growth regulator G3 factor, and myrtucommulone E. Myricetin was able to inhibit reactive oxygen species production on zymosan-stimulated whole blood phagocytes (IC_50_ = 1.6 μg/mL), in a dose-dependent manner. The J774.2 cells treated with phorbol 12-myristate 13-acetate (stimulant used to distinguish the activity of the oxidative burst from zymosan activation that is involved in phagocytosis) alone (positive control) or in combination with myrtucommuacetalone and myricetin for 30 min, showed that these compounds were able to inhibit the production of reactive oxygen species. Myricetin was able to inhibit the production of these reactive species in both stimulant processes, suggesting that this flavonoid is able to inhibit reactive oxygen species, mainly superoxide, by a myeloperoxidase independent pathway [143]. Myrtucommuacetalone and growth regulator G3 factor also inhibited the production of nitric oxide in mouse macrophages (82.3% and 59.36%, respectively), at 25 μg/mL concentration [143].

#### 3.5. Complexity of Myrtle Extracts on the Antioxidant Activity

As aforementioned, several methods have been used for evaluating the antiradicalar activity and distinct results were obtained due to diverse factors. The substrates were complex mixtures that could also contribute for the diversity of results. Sanna et al. [144] compared two spectroscopic methods (ultraviolet-visible spectroscopy and electron paramagnetic resonance) on the antioxidant ability of myrtle leaf exctracts. In both cases, DPPH free radicals were used for evaluating the antiradicalar activity of extracts, in which the depletion of DPPH in the presence of an antoxidant is measured. Since the samples used in both assays were the same, all differences in the results could only be attributed to the method itself [144]. The results showed that for ultraviolet-visible spectroscopy method there was not proportionality between the extract concentration and absorbance, because for increasing extract concentrations, the colour changed from dark purple (DPPH solution colour) to dark brown, although the extract had been depleted all the DPPH present in the solution. However, the decrease in DPPH signal intensities measured by electron paramagnetic resonance was concentration dependent. The authors concluded that the estimation of radical scavenging ability performed by electron paramagnetic resonance is more trustworthy than ultraviolet-visible spectroscopy measurements. Though this conclusion, the utilization of the ultraviolet-visible spectroscopy for DPPH method is still largely used.

The complexity of sample matrix makes difficult to assign the biological activity to a compound or to a set of compounds. However, Romani et al. [145] were able to assign the antiradical activity of myrtle leaf, from Italy, to gallotannins which were predominant in myrtle extracts as well as in the commercial extract of chestnut bark used as reference. However the amounts of gallotannins varied significantly from one harvesting year to another harvesting year (Table 3), which makes difficult to have a final product with identical magnitude of activity. The establishement of standardized extracts is required to prevent such variability. The chemical composition of tannin aqueous and hydro-alcoholic extracts of myrtle leaf (Table 3) was evaluated by HPLC/DAD/ESI-MS methods.

#### 3.6. Antioxidant Activity of the Leaf Foods

The capacity of myrtle extracts to retard food oxidation was evaluated by Turhan et al. [146] when the brining process of anchovies with sodium chloride (15 g/100 mL) was done with myrtle, rosemary and nettle extracts and stored at 4 °C, for 28 days. The lipid oxidation was followed by determining the peroxide value, thiobarbituric acid reactive substance (TBARS), and oxidative rancidity score. Myrtle and rosemary extracts were the most effective in slowing down the lipid oxidation because they decreased the peroxide value from 37.77 meq O_2_/kg, in the control, to 11.48 meq O_2_/kg, in rosemary extract; this value is not significantly different from the myrtle extract. These values were found after 28 days of storage. These extracts were also able to decrease the TBARS values from 1.89 mg MDA/kg (control) to 0.59 mg MDA/kg and 0.50 mg MDA/kg, in the case of brined anchovies with myrtle and rosemary extracts, after 28 days of storage. These values can be attributed to the highest amounts of total phenols in the myrtle (72.4 mg/g) and rosemary (52.6 mg/g) extracts, when compared to the lowest amounts of phenols in the nettle extract (7.2 mg/g). The capacity for scavenging the DPPH free radicals and the reducing power were also better for those extracts, which may explain the best capacity of myrtle and rosemary extracts for retarding the lipid oxidation of brined anchovies when stored during 28 days [146].

The application of myrtle extracts in food to prevent oxidation was also evaluated by Dairi et al. [147]. The nutritional quality of extra virgin olive oil can be lost when lipids oxidation occur, by losing its phenolic compounds, particularly during heating procedures. Due to the antioxidant properties of myrtle extracts, Dairi et al. [147] studied the effect of myrtle extract, obtained by two different methods (microwave-assisted extraction and maceration) on the preservation of nutritional quality of extra virgin olive oil, particularly of the phenolic compounds after heating processes (butane-air flame, oven and microwave). The evolution of the phenolic compounds content was monitoring by reversed phase dispersive liquid–liquid microextraction (RP-DLLME)-HPLCDAD-FLD method. The results showed that the addition of myrtle extracts not only preserved the endogenous phenolic compound of extra virgin olive oil (hydroxytyrosol, tyrosol, luteolin, apigenin, and secoiridoid 1) when compared with the control, in which myrtle extract was not added, but also reduced the specific extinction coefficient (K_232_) values. This parameter checks the degree of a vegetal oil oxidation, being indicative of the formation of primary products of oxidation. However, these benefits induced by myrtle extract, mainly constituted by galloyl quinic acid, gallic acid, and myricitrin, were dependent on the type of heating of olive oil. In the phenol preservation, the most protective effect of the myrtle extract was found during flame and microwave heating, whereas in the prevention of primary oxidation products the most effective one occurred when sample oils were submitted to the flame heating. The myrtle extract did not exert any beneficial effect on the prevention of the formation of secondary products of oxidation, that is, it did not reduce the specific extinction coefficient (K_270_) [147]. According to the authors, myrtle extracts may be a tool for improving the oxidative stability of olive oil, by improving its phenol composition.

After knowing that the enriched olive oil with myrtle extract prevented its oxidation [147], later on, Dairi et al. [148] wanted to know if the enriched oil would have better antioxidant properties acting against free radical attacks that can occur during lipid digestion. To reach the objectives, the authors studied the effect of myrtle extract, obtained by two different methods (microwave-assisted extraction and maceration), on egg yolk phosphatidylcholine/bile salts aqueous dispersion oxidation under simulated intestinal conditions (pH 7.4), that is, a model that would permit to know if such extracts could prevent lipid peroxidation that might occur in small intestine during lipids digestion. AAPH (2,2′-azobis (2-aminopropane) dihydrochloride) or a Fe^3+^/ascorbic acid system were used to initialize the phospholipid peroxidation. In addition, the capacity for preventing DPPH and peroxyl radicals (ORAC) was also checked by the authors [148]. The phenolic composition of myrtle extracts is presented in Table 3, and the chemical composition was not greatly different in both extraction procedures. The extra virgin olive oil enriched with myrtle extract increased the neutralization of DPPH and peroxyl radicals, even better than the references α-tocopherol and butylated hydroxytoluene (BHT). When the lipidic model was used, the phospholipid stability increased by a factor of 33.6% and 34.8%, for myrtle microwave assisted extraction and maceration extraction when compared to the control (without myrtle extraction), when the lipid induction was performed with the Fe^3+^/ascorbic acid system. However, when the induction was made with AAPH, the effect was very poor. This work allowed the authors to conclude that the capacity of extra virgin olive oil enriched with myrtle extract to inhibit phospholipid peroxidation under simulated intestinal conditions can be seen as a potential functional food [148].

The antioxidant activity of sea salts flavoured with Mediterranean herbs (myrtle, rosemary, and mixtures) was evaluated by Rosa et al. [122] in chemical models of lipid peroxidation and in cell cultures. Simultaneously, the authors compared the antioxidant activity of these samples with those of normal salt. The flavoured myrtle added to salt was constituted by a mixture of extract of myrtle berry juice, leaves, and myrtle essential oil. These flavoured salts preserved liposomes from Cu^2+^-induced oxidation, decreasing the accumulation of malondialdehyde, by scavenging peroxyl radicals or chelating Cu^2+^ at the aqueous phase or at the liposome particle surface/core [122]. The methanolic extracts of flavoured salts also significantly reduced the reactive oxygen species generation in *tert*-butylhydroperoxide-induced intracellular Caco-2 cells. According to the authors this ability was correlated to the capacity of extract components to permeate cell membrane and scavenge reactive oxygen species inside cells [122].

Liposomes, resembling cell membranes, are lipid molecules that can encapsulate biologically both hydrophilic and lipophilic active substances or used as lipid substrate for evaluating the capacity of samples to preventing lipid peroxidation [54]. These authors evaluated the antioxidant ability of methanolic extracts obtained from the aerial parts of Greek myrtle before and after encapsulation. The activity was followed by three methods (Rancimat method, oxidative stability by DSC, and formation of malondialdehyde). The two first methods are based on the generation of volatiles and thermal release, respectively, indicating a terminal oxidation process, whereas the generation of malondialdehyde occurs at lower temperatures and at a different stage of oxidation. The encapsulation of the extract, for the same concentration, enhanced the antioxidant action more than the same extract in pure form [54]. Therefore, the encapsulation altered the activity of extract, improving it.

#### 3.7. Anti-Inflammatory Activity of Leaf Extracts

Zaidi et al. [149] studied the effect of 24 selected Pakistani medicinal plants, including *M. communis*, which are traditionally prescribed for gastro-intestinal disorders. *Helicobacter pylori* infection is associated with gastritis, peptic ulcer, and gastric cancer. In these disorders oxidative stress is many times involved and, consequently, inflammatory processes. For this reason, the authors [149] evaluated the effect of plant extracts, such as aqueous myrtle extract, on the inhibition of secretion of IL-8 and inhibition or prevention of generation of reactive oxygen species (ROS) in clinically isolated *Helicobacter pylori* strain (193)-infected cells (human gastric cancer cell line AGS), in order to confirm the anti-inflammatory and cytoprotective effects in gastric epithelial cells attributed to those 24 plants. The authors observed that only four extracts (*Cinnamomum cassia*, *Myrtus communis*, *Syzygium aromaticum*, and *Terminalia chebula*) manifestly inhibited IL-8 secretion at both 50 and 100 μg/mL. In addition, only *Achillea millefolium*, *Berberis aristata*, *Coriandrum sativum*, *Foeniculum vulgare*, *Matricaria chamomilla*, and *Prunus domestica* were able to significantly suppression ROS generation (particularly superoxide anion radicals) from *Helicobacter pylori*-infected cells [149]. The ROS measurement generated was done by detecting the fluorescence emission, by flow cytometry, caused by the intercalation of oxidized hydroethidine, by superoxide, into DNA [59]. The chemical composition of the extracts was not determined by the authors and the results obtained were explained according to previous results. As so, they attributed the property for inhibiting IL-8 by myrtle extracts to the possible presence of myrtucommulone in the extract.

The anti-inflammatory activity of the aqueous and ethanolic extracts obtained from the aerial parts of *M. communis* was evaluated by Hosseinzadeh et al. [91] using xylene-induced ear oedema and a cotton pellet test, in mice. Antinociceptive activity was also performed using hot plate and writhing tests in mice. The ethanolic (0.05 g/kg) and aqueous extracts (0.005, 0.015, and 0.03 g/kg) demonstrated anti-inflammatory effects against chronic inflammation, whereas in the acute inflammatory activity (xylene-induced ear oedema study), the aqueous extract at doses 0.1, 0.2, and 0.03 g/kg showed significant anti-inflammatory activity. The ethanolic extract also had activity against acute inflammation in all doses (0.05, 0.15, and 0.35 g/kg), but was not dose-dependent. The aqueous and ethanolic extracts of the aerial parts of myrtle exhibited antinociceptive activity. The authors suggested that this activity might be mediated by opioid receptors [91]. The chemical composition of the extracts was not performed but the authors attributed the antinociceptive and anti-inflammatory activities to flavonoids and/or tannins, according to the references consulted by them. The highest concentrations of extracts with anti-inflammatory and antinociceptive activities were lower than the LD_50_ (lethal dose 50) values found for aqueous and ethanolic extracts (0.473 and 0.79 g/kg, respectively) [91].

Generally, the antioxidant and anti-inflammatory activities of myrtle extracts have been attributed to the phenolic compounds. The higher activity of leaf extracts than berry extracts were attributed by some authors to the presence of hydrolysable tannins in leaf extracts at higher concentrations than in berry extracts, which means the weak influence of anthocyanins, present in coloured berries, in the antioxidant activity [103,135]. However, Feisst et al. [150] reported, for the first time, that two nonprenylated acylphloroglucinols, myrtucommulone and semimyrtucommulone, isolated from leaf extracts of myrtle, potently suppressed the biosynthesis of eicosanoids by inhibiting cyclooxygenase-1 (COX-1) and 5-lipoxygenase (5-LOX) *in vitro* and *in vivo* at IC_50_ values ranging from 1.8 to 29 μM. These enzymes are involved in the formation of the proinflammatory prostaglandins and leukotriens, respectively. At the same time, the authors showed that myrtucommulone and semimyrtucommulone were able to prevent the mobilization of Ca^2+^ in polymorphonuclear leukocytes at IC_50_ = 0.55 μM and 4.5 μM, respectively, mediated by G protein signaling pathways. This effect inhibited the generation of reactive oxygen species (peroxide) and the release of elastase at similar concentrations. However, the phenolic part of those acylphloroglucinols (isobutyrophenone) was much less effective or even not active [150]. However, the acylphloroglucinols only inhibited partially peroxide formation or failed to inhibit elastase release when ionomycin was added to polymorphonuclear leukocytes. According to these results, the authors suggest that the suppression of Ca^2+^ mobilization by the myrtle acylphloroglucinols is the main cause for the inhibition of peroxide formation and elastase release induced by fMLP (*N*-formylmethionyl-leucyl-phenilalanine), which is confirmed when ionomycin is added to the system that needs much higher concentrations of the acylphloroglucinols to produce biological effect. Ionomycin did not use the G protein signaling pathways for elevating internal Ca^2+^ [150].

The inhibition effect of myrtucommulone on COX-1 in human platelets, and 5-LOX in intact polymorphonuclear leukocytes, observed by Feisst et al. [150] led Koeberle et al. [151] to ascertain if this acylphloroglucinol was able to inhibit selectively prostaglandin E_2_ (PGE_2_) via microsomal PGE_2_ synthase (mPGES)-1. For this purpose, the authors measured the effect of myrtucommulone in diverse systems (cell-free assay using microsomal preparations of interleukin-1β-stimulated A549 cells as the source of mPGES-1; intact A549 cells, and lipopolysaccharide stimulated human whole blood). The results observed by the authors were that myrtucommulone was able to inhibit the conversion of PGH_2_ to PGE_2_ (IC_50_ = 1 mmol/L) in the cell-free mPGES-1 system. In addition, the levels of PGE_2_ also diminished in intact A549 cells and in human whole blood at low micromolar concentrations, nevertheless the inhibition of COX-2 by myrtucommulone in A549 cells or isolated human recombinant COX-2 was only observed for higher concentrations (>30 mmol/L). Concerning COX-1 inhibition, the authors observed IC_50_ > 15 mmol/L values, both in cellular or cell-free systems, that is, only presenting moderate activity. According to these results the anti-inflammatory activity of myrtucommulone is due to the suppression of PGE_2_ formation and not so much to the inhibition of the COX enzymes [151].

The anti-inflammatory activity of myrtucommulone isolated from myrtle leaves was evaluated *in vivo* by Rossi et al. [152]. In this study, the authors induced inflammation in mice by the subplantar and intrapleural injection of carrageenan, respectively, that triggers the development paw oedema and pleurisy. The action of myrtucommulone was followed by administering the compound intraperitoneally. Myrtucommulone, at concentrations 0.5, 1.5, and 4.5 mg/kg i.p., reduced the development of mouse paw oedema and, at 4.5 mg/kg i.p., 30 min before and after carrageenan, showed anti-inflammatory activity in the pleurisy model. The mechanism involved in the anti-inflammatory activity of myrtucommulone was determined by the authors and they observed that carrageenan injection in the pleurisy test reduced the exudate volume and leukocyte number, myeloperoxidase activity, the lung intercellular adhesion molecule-1 and P-selectin, tumor necrosis factor-α (TNF-α) and interleukin-1β (IL-1β), leukotriene B4 (LTB_4_), lung peroxidation (thiobarbituric acid-reactant substance), and nitrotyrosine and poly (ADP-ribose). Such results permitted to the authors to suggest that the mechanisms involved in the protection effect of myrtucommulone in lung injury include suppression of adhesion molecules, inhibition of LTB_4_ generation and neutrophil infiltration [152].

The anti-inflammatory activity of natural compounds is sometimes attributed to their capacity for protecting tissues against oxidative damage. Chronic liver disease with fibrosis is a health problem that can be caused by duct obstruction which leads to cholestasis and liver damage. The oxidative stress seems to have an important role in the damage. Sen et al. [75] aimed at investigating the antioxidant and antifibrotic effect of *Myrtus communis* extracts against against liver injury and fibrosis occurring in rats with biliary obstruction in animal models. Glutathione and superoxide dismutase values that decreased in damaged liver, and malondialdehyde levels, myeloperoxidase activity, tissue luminol, lucigenin, transforming growth factor-beta (TGF-β), and hydroxyproline levels that increase in damaged liver, the treatment with myrtle extract reversed all of these parameters, that is, this extract protects the liver tissues against oxidative damage through its radical scavenging and antioxidant activities, decreasing the fibrotic activity by reducing the hepatic TGF-β and hydroxyproline contents [75]. Later on, Sen et al. [74] reported that ethanolic extracts obtained from myrtle leaves when supplemented to the diet of Wistar albino rats in which colitis was induced by acetic acid, on the fourth day, they decreased the levels of malondialdehyde, tissue luminol, lucigenin, nitric oxide, and peroxoxynitrite chemiluminescence, as well the myeloperoxidase activity, and increased the glutathione levels, when compared to those animals in which the disease was induced and any myrtle supplementation was given. The study showed that ethanol extract had significant antiiinflammatory activity protecting the tissues against oxidative damage [74].

Fekri et al. [89] evaluated the biochemical and histopathological effect of preventive and therapeutic doses of extracts of myrtle leaves against bleomycin-induced pulmonary fibrosis in an animal model. Inflammatory and oxidative processes are involved in the pulmonary fibrosis. For this reason, the authors [89] evaluated the effect of myrtle extract on the lipid peroxidation as well as on the activity of catalase in the animal submitted to bleomycin. The oxidation of lipids decreased and the activity of catalase increased, that is, the oxidative stress promoted by bleomycin was reversed with the myrtle treatment. Simultaneously, the methanolic extract of myrtle leaves decreased the hydroxyproline concentration in animals subjected to bleomycin-induced pulmonary fibrosis in early and late phases. Hydroxyproline is an indicator of collagen deposition in lungs in pulmonary fibrosis. The authors also reported the improvement in inflammation and fibrosis in myrtle group [89].

The anti-inflammatory activity of the aqueous and ethanolic extracts obtained from the aerial parts of *M. communis* was evaluated by Hosseinzadeh et al. [91] using xylene-induced ear oedema and a cotton pellet test, in mice. Antinociceptive activity was also performed using hot plate and writhing tests in mice. The ethanolic (0.05 g/kg) and aqueous extracts (0.005, 0.015, and 0.03 g/kg) demonstrated anti-inflammatory effects against chronic inflammation, whereas in the acute inflammatory activity (xylene-induced ear oedema study), the aqueous extract at doses 0.1, 0.2, and 0.03 g/kg showed significant anti-inflammatory activity. The ethanolic extract also had activity against acute inflammation in all doses (0.05, 0.15, and 0.35 g/kg), but was not dose-dependent. The aqueous and ethanolic extracts of the aerial parts of myrtle exhibited antinociceptive activity. The authors suggested that this activity might be mediated by opioid receptors [91]. The chemical composition of the extracts was not performed but the authors attributed the antinociceptive and anti-inflammatory activities to flavonoids and/or tannins, according to the references consulted by them. The highest concentrations of extracts with anti-inflammatory and antinociceptive activities were lower than the LD_50_ (lethal dose 50) values found for aqueous and ethanolic extracts (0.473 and 0.79 g/kg, respectively) [91].

One problem of the plant extracts is the heterogeneity in the concentration of the bioactive compounds. This fact may originate biological responses with different strength. For overcoming this problem, there is the possibility to obtain standardized extracts with well defined concentrations of the bioactive compounds. Fiorini-Puybaret et al. [153] used a standardized ethanolic extract (0.75% of myrtucommulones) obtained from myrtle leaves, with the trade name Myrtacine^®^ (Ducray Laboratory, Lavaur, France) with the objective to ascertain if this extract is able to treat acne in its diverse aspects: antibacterial activity against *Propionibacterium acnes*; antiproliferative activity on human keratinocytes and anti-inflammatory properties using a cellular model of inflammation. These biological properties were also compared with myrtucommulones A and B’, which are present in the standardized extract. This approach was based on the fact that the nonprenylated acylphloroglucinols of myrtle possessed antimicrobial, antioxidant, and anti-inflammatory activities [30,150,154]. Anti-inflammatory effect was determined through the measurement of 6-keto-prostaglandin F1α and [3H]-arachidonic acid metabolite production by A23187-stimulated human keratinocytes. COX and lipoxygenase LOX metabolite production from ionomycin-stimulated human keratinocytes was also evaluated as well as the lipase activity [153]. The results showed that A23187-stimulated keratinocytes in the presence of Myrtacine^®^ at 10 μg/mL inhibited in 23% the production of 6-keto PGF1α compared to the control. Preincubation of SVK14 keratinocytes with 0.1 and 0.5 μg/mL myrtucommulone A also significantly reduced 6-keto PGF1α production in 21% and 17%, respectively, whereas myrtucommulone B’ only lowered the production of 6-keto PGF1α in 9%. At 3 and 10 μg/mL, Myrtacine^®^ significantly decreased all metabolite production from cyclooxygenase (6-keto PGF1α, PGE2, PGF2α, PGD2, and PGA2) and lipoxygenase (LTB4 and 12-hydroxyeicosatetraenoic acid or HETE). Moreover, Myrtacine^®^ also exhibited anti-lipase activity: at 100 μg/mL and 1 mg/mL was able to inhibit the activity of lipase by 53% and 100%, respectively [153]. This anti-inflammatory activity along with the anti-*P. acnes* and antiproliferative activities can lead Myrtacine^®^ to be used in the treatment of comedones and inflammatory acne lesions [153].

The mechanisms involved in the anti-inflammatory activity of myrtucommulone isolated from myrtle leaves have been cleared by diverse research teams as aforementioned; nevertheless there is no information about its bioavailability. Gerbeth et al. [155] proposed to study the metabolic stability of that acylphloroglucinol, obtained by synthesis, using rat and human liver microsomes and its oral availability in a pilot rat study. The study started by using Caco-2 cells and the results showed a high absorption of myrtucommulone. In rat model, the authors [155] reported that after 1 h of administrating 4 mg/kg myrtucommulone, an average plasma level of 258.67 ng/mL was observed. Physiologically-based pharmacokinetic modelling of myrtucommulone in the rat, it was observed that it was rapid and extensively distributed by plasma, skin, muscle, and brain. Moreover, myrtucommulone undergoes phase I biotransformation in human and rat liver microsomes, resulting hydroxylated and demethylated metabolites [155].

## 4. *Myrtus nivellei*

The antioxidant and anti-inflammatory activities of berries and/or leaves of *M. nivellei* are much fewer reported than those found for *M. communis*. Rached et al. [156] collected fifty two plants in different regions of Algeria and evaluated their antioxidant activity through two methods: DPPH and β-carotene-linoleic acid bleaching. Forty-eight active extracts were found from 38 Algerian species and *M. nivellei* leaves were in this group with IC_50_ value = 4.90 μg/mL (DPPH assay), in an aqueous extract, obtained by decoction, with total phenol concentration of 242.68 mg/gallic acid equivalent/g and total flavonoid content of 28.53 mg catechin equivalent/g. The IC_50_ value was close to that found for BHA (IC_50_ = 4.15 μg/mL) but higher and, therefore, poorer than quercetin (IC_50_ = 1.66 μg/mL) and ascorbic acid (IC_50_ = 2.66 μg/mL). A liquid–liquid fractionation assay from the aqueous extract was performed using solvents with increasing polarity (chloroform, ethyl acetate and *n*-butanol). In the DPPH assay, the IC_50_ values found for these extracts were: 53.50, 3.08, and 4.40 μg/mL, respectively. The remaining aqueous extract also had capacity for scavenging the DPPH free radicals (IC_50_ = 64.84 μg/mL). A good linear correlation was found between the antioxidant activity of the diverse fractions and total phenols and total flavonoids’ contents [156].

Different extracts of leaves of *M. nevellei* were obtained (aqueous, ethanolic, and methanolic) and their antioxidant activities were evaluated by [157]. Ethanolic extract revealed to be the most effective for scavenging DPPH free radicals (EC_50_ = 0.59 μg/mL), closest to the reference, ascorbic acid (EC_50_ = 0.39 μg/mL). However, the aqueous extract more easily reduced ferric ion (64.86%) than the ethanolic extract (35.14%). The ethanolic extract possessed higher amounts of phenols (734.3 μg gallic acid equivalent/mg) and flavonoids (181.1 μg quercetin equivalent/mg) than the aqueous extract (466.5 μg gallic acid equivalent/mg and 135.5 μg quercetin equivalent/mg, respectively) [157].

The anti-inflammatory activity of methanolic extract of the aerial parts of *M. nivellei* was evaluated for the first time by Touaibia and Chaouch [158] using the carrageenan-induced paw oedema test. The authors revealed that the dose of 400 mg/kg was able to reduce significantly the paw oedema (80.41%). This inhibition percentage was similar to that of diclofenac, but at 50 mg/kg. Oral lethal dose 50 (LD_50_) of the methanolic extract was higher than 1000 mg/kg, and therefore, the authors considered this dose as being highly safe [158].

*M. nivellei* is a Sahara-endemic plant used in folk medicine. In the absence of any preservation programmes can originate its disappearance very quickly. Touaibia and Chaouch [19] obtained in vitro calli from this species, evaluated their antioxidant activity and compared to those obtained from leaf extracts. Total phenol and total flavonoid’ contents of calli extracts were inferior (73 μg gallic acid equivalent/g and 91 μg quercetin equivalent/g) when those obtained from leaf extracts (in situ): 348 μg gallic acid equivalent/g and 152.25 μg quercetin equivalent/g. These lower amounts of phenols might be responsible for the lowest capacity of calli for scavenging the DPPH free radicals (EC_50_ = 1.44 mg/mL). EC_50_ value for leaf samples (in situ) was 0.98 mg/mL. Nevertheless, calli extract exhibited higher capacity for preventing lipid peroxidation and reducing power than leaf extracts [19].

The chemical composition of Saharan myrtle only very recently was unravelled [20,22]. The biological properties of these extracts and their compounds were also reported [20]. From leaves of *M. nivellei*, Mansour et al. [20] obtained aqueous extracts by decoction and infusion that were analysed by ultrahigh-performance liquid chromatography photodiode array high-resolution mass spectrometry (UHPLC-PDA-HRMS) and then confirmed by nuclear magnetic resonance (NMR) spectroscopy. The phenolic compounds present in the infusion and decoction were also quantified by HPLC-UV-PDA. The fourteen compounds identified are depicted in Table 4. Myricetin 3-*O*-β-d-(6″-galloyl)glucopyranoside, isomyricitrin, and myricitrin were the major compounds present in the aqueous extracts of *M. nivellei*. Decoction extracted more phenols (150.5 mg/g) than the infusion (102.6 mg/g), corresponding to 73.8 and 23.6 mg/100 mL of a single tea cup, respectively. The capacity of decoction, infusion, and isolated compounds for scavenging the DPPH free radicals were analysed by the authors [20] and compared with green and black teas. The EC_50_ values for decoction and infusion of black and green teas were: EC_50_ = 10.2, 18.6, 22.9, and 18.0 μg/mL, respectively. The activities of isolated compounds were better than those of infusion and decoction. The EC_50_ values for 3,4,5-*tri*-*O*-galloyl-quinic acid, myricetin-3-*O*-β-d-(6″-galloyl)glucopyranoside, *iso*myricitrin, 1,2,3,6-tetra-*O*-galloyl glucose, myricitrin, quercetin-3-*O*-β-d-(6″-galloyl)glucopyranoside, myricetin-3-*O*-β-xyloside, myricetin, and quercitrin were: 3.8, 5.6, 6.7, 4.0, 4.3, 8.8, 7.0, 3.5, and 5.9 μg/mL, respectively. Myricetin, 3,4,5-tri-*O*-galloyl-quinic acid and 1,2,3,6-tetra-*O*-galloyl glucose had a similar capacity for scavenging the free radicals of that of the reference, ascorbic acid, that is a powerful natural soluble antioxidant. These results indicate that the effective antioxidant capacity of *M. nivellei* teas can be attributed to flavonoids, their glycosides and polygalloyl derivatives [20]. Nevertheless, it seems that when in combination with the other phenolic compounds, some antagonism may occur among them, since the antioxidant activity of the whole tea is lower than the isolated compounds.

The chemical composition of crude aqueous extract, ethyl acetate and butanol fractions of Saharan myrtle leaves conducted by liquid chromatography with diode array detection, coupled to mass spectrometry (ion trap) with electrospray ionization (HPLC-DAD−ESI/MS*n*) permitted to identify 17, 25, and 19 compounds, respectively (Table 4) [22]. The ethyl acetate fraction had the highest concentration of phenol compounds, followed by the butanol fraction and the crude aqueous extract. The antioxidant activities of the extracts were evaluated through three methods: DPPH, reducing power, inhibition of β-carotene bleaching, and thiobarbituric acid reactive substance methods. The results showed that the ethyl acetate fraction exhibited better activity than the remaining extracts or fraction. In the DPPH method, the EC_50_ values found for ethyl acetate, butanol fractions and crude aqueous extracts were: 3.27, 4.6, and 7.1 μg/mL, respectively; in the reducing power, the EC_50_ values were: 3.15, 3.93, and 6.23 μg/mL, respectively; in the inhibition of β-carotene bleaching, EC_50_ values were: 82, 92.9, and 112 μg/mL, respectively; and in the thiobarbituric acid reactive substance, the EC_50_ values were: 0.46, 0.74, and 0.87, respectively. According to the authors, the best activity of the ethyl fraction can be attributed to the presence of some compounds, such as myricetin-hexosyl-gallate, myricetin-3-*O*-rhamnoside, gallocatechin-gallate-dimer, digalloyl, trigalloyl-HHDP-glucoside, tetragalloylglucoside, and of the quercetin and kaempferol derivatives. Such results are in line with those already reported by Pereira et al. [111,112] in which the authors found a good correlation between antioxidant activity and flavonol glycosides in *M. communis* extracts. With the exception of the inhibition of β-carotene bleaching method, all samples had better activity than the reference used, Trolox. The anti-inflammatory activity was measured through the capacity of samples to suppress the NO production by LPS (lipopolysaccharide)-induced murine macrophage-like RAW 264.7 cells. Such as observed for the antioxidant activity, ethyl acetate was also the best sample (EC_50_ = 104 μg/mL) for suppressing NO production and, therefore, best anti-inflammatory activity, followed by the butanol fraction (EC_50_ = 127 μg/mL) and the crude aqueous sample (EC_50_ = 149 μg/mL), however all of them presenting much lower activity than the reference, dexamethasone (EC_50_ = 16 μg/mL). The authors also attributed the anti-inflammatory activity to the presence of flavonols, ellagitannins and phenolic acids. The authors [22] also evaluated the cytotoxic properties of Saharan myrtle extract and fraction against diverse tumor cell lines (breast cancer MCF-7, lung cancer NCI-H460, cervical cancer HeLa, and liver cancer HepG2 lines). The ethyl acetate fraction showed a significant higher potential against all cancer cell lines, followed by the butanol fraction and the crude aqueous extract, nevertheless the same fraction was also that exhibited the lowest cytotoxicity on non-tumor cells (porcin liver primary cells, PLP2). According to the authors [22], ellagic acid, ellagitannins, quercetin, and its derivatives may have a crucial role in the cytotoxicity activity.

## 5. Conclusions

The myrtle berries are mainly used for doing liqueur; nevertheless berries can also be used for making jam, preserving their biological properties, such as the liposome oxidation. The antioxidant activity of berries, due to the presence of phenols, also seems to be a possibility to use the berries’ pulp as prebiotics in some food formulations, such as probiotic-enriched ice-creams. For a myrtle liqueur of high quality it is also required that berries must be processed immediately after harvest. Two approaches can be followed: to store fruits in adequate conditions, such as controlled atmospheres; or to process the berries and store the hydro-alcoholic extracts. For the former case, the results showed that berries held at 80% O_2_ at 2 °C preserve their quality of phenolic and anthocyanins contents, and antioxidant activity, for at least 20 days. When fruits are immediately submitted to maceration, the extract obtained is stable for three months, being flavonoids and, particularly, anthocyanins the most instable compounds. The antioxidant activity of berry extracts can only be poorly attributed to the anthocyanins, since white berries, in some cases, exhibit stronger antioxidant activity than dark blue berries. Some type of flavonoids and gallic acid and their derivatives may be responsible for the antioxidant activities found in berry extracts. Beyond the maceration, several other methods of extraction (e.g., supercritical fluid extraction, ultrasound-assisted extraction, and decoction) may be used, nevertheless did not provide much better phenol content or antioxidant activity. The type of solvent of solvent mixtures used revealed also to be important on the capacity for extracting higher amounts of some types of phenol compounds and, therefore, on the antioxidant activity, nevertheless sometimes the results are dissimilar, depending on the research team and conditions of work. Seeds revealed to be best antioxidants than the remaining parts of the fruit, probably due to the presence of higher concentrations of galloyl derivatives. When leaf and berry extracts of myrtle were compared in terms of antioxidant capacity, leaf extracts revealed to be those that exhibited higher antioxidant activity, not only to the highest amounts of total phenols but also for the highest concentrations of hydroxybenzoic acids and flavonols and their derivatives although the most important factor in the relevant activity of leaf myrtle extracts is the ratio between the sum of galloylglucosides, ellagitannins, and flavonols and also of the ratio between these galloyl derivatives and galloyl-quinic acids. The utilization of leaf extracts for stabilizing complex lipid systems, olive oil, and brined anchovies revealed to be possible, particularly when myricetin 3-*O*-rhamnoside is present, due to its antioxidant activity. The anti-inflammatory activity was also reported for both berry and leaf extracts of myrtle. In some cases such activity was attributed to the flavonoids and/or hydrolysable tannins, nevertheless nonprenylated acylphloroglucinols (e.g., myrtucommulone and semimyrtucommulone) were revealed to have also a remarkable role in that activity.

The chemical composition and antioxidant activity of Saharan myrtle is much less studied, most likely to its restricted distribution, which only appears in specific places of the Sahara. Only very recently, a detailed chemical composition of their extracts was performed as well as their antioxidant, anti-inflammatory, cytotoxic, and antibacterial activities. In the aerial parts or leaves were possible to find compounds belonging to the galloyl derivatives, flavonols, and flavonols derivatives, and phenolic acids as reported for myrtle extracts, nevertheless, some new compounds were found, such as 2-hydroxy-1,8-cineole-β-d-glucopyranoside, 2-hydroxy-1,8-cineole 2-*O*-α-l-arabinofuranosyl (1→6)-β-d-glucopyranoside, rugosin A, rugosin B, and valoneic acid dilactone, which were not reported in myrtle extracts. The effective antioxidant capacity of *M. nivellei* teas can be attributed to flavonoids, their glycosides, and polygalloyl derivatives.

## Figures and Tables

**Table 1:** Biological properties attributed to *Myrtus communis* (antioxidant and anti-inflammatory activities are not included).

Plant Part Used	Compounds	Biological Properties	References
		Antimicrobial	
Leaves	Not reported	Bacterial vaginosis	[23]
Leaves	Not reported	*Propionibacterium acnes*	[24]
Leaves and berries	Not reported	- Spoilage bacteria *Pseudomonas aeruginosa* IH*, Pseudomonas aeruginosa* CECT 118*, Pseudomonas aeruginosa* CECT 110T*, Pseudomonas fluorescens* CECT 378 and *Bacillus subtilis* DCM 3366 - Food-borne pathogenic bacteria, namely *Escherchia coli K12, Listeria innocua* CECT 4030*, Listeria monocytogenes* CECT 4032*, Enteroccus faecium CECT 410, Staphylococcus aureus* MBLA, *Staphylococcus aureus* CECT 976, *Staphylococcus aureus* CECT 794 and *Proteus vulgaris* CECT 484	[25]
Leaves	Not reported	One hundred and twenty strains of *Escherichia coli* isolated from the urine culture	[26]
Leaves	Not reported	*Streptococcus pneumoniae*, *Streptococcus pyogenes*, *Streptococcus agalactiae*, *Listeria monocytogenes, Campylobacter jejuni*, *Staphylococcus aureus*, *Micrococcus luteus, Escherichia coli, Proteus vulgaris,* and *Pseudomonas aeruginosa*	[27]
Leaves	Not reported	*Aeromonas hydrophilica* isolated from four hundred and fifty samples from the intestines of the infected *Cyprinus carpio* fish	[28]
Leaves	Not reported	Ninety-six *P. aeruginosa* strains isolated from 400 burn patients (men and women) in Iranian hospital	[29]
Leaves	*Galloylated nonprenylated* *phloroglucinol glucosides:* Gallomyrtucommulone A Gallomyrtucommulone B Gallomyrtucommulone C Gallomyrtucommulone D	*Staphylococcus aureus* strain ATCC 25923 gift of E. Udo (Kuwait University, Kuwait) *S. aureus* RN4220 containing plasmid pUL5054, which carries the gene encoding the MsrA macrolide efflux protein, provided by J. Cove *S. aureus* XU-212 which possesses the TetK tetracycline efflux protein, provided by E. Udo *S. aureus* SA-1199B, which overexpresses the *norA* gene encoding the NorA MDR efflux protein, provided by G. Kaatz *S. aureus* EMRSA-15 is an epidemic strain of MRSA gift of P. Stapleton, School of Pharmacy, University of London	[30]
Seeds	Not reported	*Escherichia coli* (PTCC No. 1330), *Pseudomonas aeruginosa* (PTCC No. 1074), *P. fluorescens* (PTCC No. 1181), *Klebsiella pneumoniae* (PTCC No. 1053), *Bordetella bronchiseptica* (PTCC No. 1025), *Staphylococcus aureus* (PTCC No. 1112), *S. epidermidis* (PTCC No. 1114), *Micrococcus luteus* (PTCC No. 1170), *Bacillus cereus* (PTCC No. 1015), and *B. pumilis* (PTCC No. 1319)	[31]
Berry seeds	Hydroxybenzoic acid hexose Delphinidin-3-*O*-galactoside Delphinidin-3-*O*-glucoside Quercetin hexoside Delphinidin-3-*O*-rhamnoside Delphinidin rutinoside Delphinidin-3-(6 coumaroyl)-glucoside Petunidin-3-*O*-glucoside Petunidin diglucoside Petunidin malonylglucoside Petunidin-3-*O*-rutinoside Isorhamnetin-*O*-rhamnoside Malvidin-*O*-galactoside Malvidin-*O*-glucoside Peonidin diglucoside Petunidin methyl pentose	*Escherichia coli* ATCC 8739, *Salmonella typhimurium* NCTC 6017, *Staphylococcus aureus* ATCC 29213, *Pseudomonas aeruginosa* ATCC 27853, *Aeromonas hydrophila* EI, and *Bacillus cereus* ATCC 1247	[32]
Leaves	Not reported	*Streptococcus mutans* (PTCC 1683)	[33]
Leaves	Not reported	Gram-positive (*Listeria monocytogenes* and *Bacillus cereus*) Gram-negative (*Escherichia coli* O157:H7) bacterial strains Fungal strain (*Candida albicans*)	[34]
Leaves and berries	Not reported	*Staphylococcus aureus* (ATCC 6538), *Bacillus subtilis* (ATCC 6059), *Micrococcus flavus* (SBUG 16), *Escherichia coli* (ATCC 11229), *Pseudomonas aeruginosa* (ATCC 27853), and three multi-resistant *Staphylococcus* strains (*Staphylococcus epidermidis* 847, *Staphylococcus haemolyticus* 535, *Staphylococcus aureus* north German epidemic strain) *Candida maltosa* (SBUG)	[35]
Leaves	Not reported	*Staphylococcus aureus*, *Staphylococcus epidermidis*, *Escherichia coli*, *Bacillus subtilis* and *Serratia marcescens*	[36]
Leaves	Myrtucommulones J-L Myrtucommulone A	*Staphylococcus aureus* (ATCC 25923)	[37]
Leaves	Not reported	*Enterococcus faecalis* (ATCC 29212)	[38]
Leaves	Not reported	Bacterial vaginosis	[39]
Leaves	Not reported	*Pseudomonas aeruginosa*	[40]
Leaves	Not reported	*Microsporum canis* ATCC 32903, *M. gypseum* ATCC 14683, and *Trichophyton mentagrophytes* ATCC 1481 (var. *interdigitale*) from Tehran University of Medical Sciences	[41]
Aerial parts	Not reported	*Trichophyton mentagrophytes, T. interdigitale*, *Microsporum canis*, and *M. gypseum* (10 strain of each)	[42]
Leaves	Not reported	*Escherichia coli* O157:H7, *Yersinia enterocolitica* O9, *Proteus* spp*.*, and *Klebsiella pneumoniae*	[43]
Berries	Not reported	*Helicobacter pylori* (12 clinical isolates)	[44]
Leaves	Not reported	*Staphylococcus aureus* (489 samples) isolated either from healthy carriers (nose and throat) or clinical samples *S. aureus* used as reference strains for comparison: ATCC 25923, ATCC 9144, ATCC 29737, ATCC 12596, and Bristol A 9596	[45]
Leaves	5-Acetoxy-4-hydroxy-4-isobutyl-2,2,6,6-tetramethylcyclohexan-1,3-dione β-Sitosterol Isomyrtucommulone-B Endoperoxide-G-3-hormone Gallic acid Myricetin-3-*O*-α-l-rhamnoside Myricetin-3-*O*-β-d-glucoside Myricetin-3-*O*-β-d-galactoside-6″-*O*-gallate (8)	*Propionibacterium acnes* NRRL (B-4224)	[46]
Leaves	Myrtucommulone A	*Bacillus subtilis*, *Staphylococcus aureus*, *Escherichia coli*, *Saccharomyces cerevisiae*, *Escherichia coli B*, *E. coli* CW 3747*, E. coli* K-12*, Klebsiella pneumoniae*, *Proteus mirabilis*, *Proteus morganii*, *Shigella dysenteriae*, *S. flexneri, Salmonella typhimurium*, *Pseudomonas fluorescens*, *Vibrio cholerae*, *Serratia*, *Staphylococcus aureus*, *S. albus*, *Bacillus subtilis* W23, *B. subtilis* 16, *B. pumilus*, *Streptococcus faecalis*, *Corynebacterium diphtheriae*, and *C. xerosis*	[47]
Leaves	Not reported	*Helicobacter pylori*	[48]
Leaves	Myrtucomvalones A–C Callistiviminene J-N	Respiratory syncytial virus (RSV)	[49]
Aerial parts	Myrtucommulone B-E Usnone A Tectochrysine 2,5-Dihydroxy-4-methoxybenzophenone (cearoin) β-Sitosterol Sideroxylin Ursolic acid Corosolic acid Arjunolic acid Erythrodiol Oleanolic acid Betulin	*Escherichia coli*, *Bacillus subtilis*, *Shigella flexneri*, *Staphylococcus aureus*, *Pseudomonas aeruginosa*, and *Salmonella typhi*	[50]
Leaves	Semimyrtucommulone myrtucommulone A	*Staphylococcus aureus* strains RN4220 (Msr(A)), XU212 (Tet(K)), 1199-B (Nor(A)), and ATCC 25923	[51]
Leaves	Myrtucommunins A-D 6-Methyl-isomyrtucommulone B 4-Methyl myrtucommulone B 2-Isobutyryl-4-methylphloroglucinol 1-*O*-β-d-glucopyranoside Chromone derivative, undulatoside A 6’-*O*-gallate	*Escherichia coli*, *Staphylococcus aureus* (MRSA), *Staphylococcus aureus* (MSSA), and *Bacillus subtilis*	[52]
Leaves	Silver nanoparticles synthesized using *Myrtus communis* L. leaf extract	*Escherichia coli*, *Bacillus subtilis*, *Pseudomonas aeruginosa*, *Staphylococcus aureus* methicillin-resistant, *Staphylococcus aureus*, and *Enterococcus faecalis*	[53]
Leaves	Before and after encapsulation in liposomes	*Staphylococcus aureus* (ATCC25923), *Staphylococcus epidermidis* (ATCC 12228), *Staphylococcus mutans* (ATCC 31989) and *Staphylococcus viridans* (ATCC 19952), *Pseudomonas aeruginosa* (ATCC 27853), *Escherichia coli* (ATCC 25922), *Enterobacter cloacae* (ATCC 13047) and *Klebsiella pneumoniae* (ATCC13883), *Candida albicans* (ATCC 10231), *Candida tropicalis* (ATCC 13801) and *Candida glabrata* (ATCC 28838), and *Listeria monocytogenes*	[54]
		Other organisms	
Leaves	Not reported	Anti-*Leishmania tropica* on an *in vitro* model	[55]
Aerial parts	Not reported	*In vivo*, anti- *Plasmodium berghei* in female Swiss albino mice, weight 18–20 g *In vitro*, chloroquine-sensitive strain (3D7) of *P. falciparum*	[56]
Not reported (myrtle was obtained from a local grocery for herbal plants)	Not reported	Induced programmed cell death in hydatid cyst protoscolices	[57]
Aerial part	Not reported	*In vitro*, anti-chloroquine-resistant (K1) and chloroquine-sensitive (3D7) strains of *Plasmodium falciparum* *In vivo*, anti-*Plasmodium berghei* infection in adult male albino mice	[58]
		Cytotoxicity	
Leaves	Not reported	Cytotoxic activities against J774 cells (Mouse BALB/c monocyte macrophage)	[55]
Leaves and berries	Not reported	Cytotoxic activities against urinary bladder 5637 and human breast carcinoma MCF-7 cell lines	[35]
Leaves	Myrtucommulones J-L Myrtucommulone A	Cytotoxic activities against human haematological tumor cell line MT-4. Cytotoxic activities against against solid tumor cell lines (HepG2 or human liver cancer, DU145 or human prostate cancer cell lines), and against “normal” human tissue cells (CRL7065)	[37]
Leaves	Myrtucommulone A	Cytotoxic activities against U-937 (human lung (lymphoblast), K-562 (human blood (chronic myelogenous leukemia), leukemic cell line KBM-5, and MEG-01 (human bone marrow) cell lines	[59]
Aerial part	Not reported	Cytotoxic activities against MCF7 (breast adenocarcinoma), HepG2 (hepatocellular carcinoma), WEHI (fibrosarcoma), and MDBK (normal kidney cells)	[58]
Not reported	Not reported	L20B (cell line a mouse cell-line genetically engineered to express human poliovirus receptor, CD155 cell lines), RD (rhabdomyosarcom), and Vero (African green monkey kidney)	[60]
Not reported	Myrtucommulone A	Mitochondrial lysates from leukemic HL-60 cells	[61]
Leaves	Myrtucommulone Semi-myrtucommulone	Jurkat-A3 cells, caspase-8-deficient Jurkat cells, FADD deficient Jurkat cells, PC-3 (androgen-independent prostate carcinoma), LNCaP (androgen-dependent prostate carcinoma), H9 (cutaneous T-cell lymphoma), DLD-1 (colorectal adenocarcinoma), HL-60 (acute promyelocytic leukaemia), Jurkat (acute T-cell leukaemia) and Jurkat DD3 (mutated in CD95), KFR (rhabdomyosarcoma) and UKF-NB-3 (neuroblastoma) cells, mono Mac 6 (MM6, acute monocytic leukaemia) cells, and human peripheral blood mononuclear cells (PBMC)	[62]
Not reported	Myrtucommulone	Mouse Breast cancer cell line 4T1, mouse embryonic fibroblasts, and human dermal fibroblasts (hDFs)	[63]
Leaves	Myrtucomvalones A–C Callistiviminene J-N	Human larynx epidermoid carcinoma cells (HEp-2) cells	[49]
Leaves	Myricetin-3-*O*-galactoside Myricetin-3-*O*-rhamnoside	Human chronic myelogenous leukemia cell line K562	[64]
Leaves	3,5-*O*-Di-galloylquinic acid	Human chronic myelogenous leukemia CML cell line K562	[65]
		Genotoxicity/mutagenicity	
Leaves	Not reported	Protective effect against genotoxicity on the SOS reponse induced by Aflatoxin B1 (AFB1) and Nifuroxazide in *Escherichia coli* PQ37	[66]
Leaves	Not reported	Protective effect against the mutagenicity induced by aflatoxin B1 (AFB1) in *Salmonella typhimurium* TA100 and TA98 assay systems, and against the mutagenicity induced by sodium azide in TA100 and TA1535 assay system	[67]
Leaves	Not reported	Protective effect against on the mutagenicity induced by aflatoxin B1 in *Salmonella typhimurium* TA100 or TA98	[68]
Leaves	Myricetin-3-*O*-galactoside Myricetin-3-*O*-rhamnoside	Protective effect against the mutagenicity induced by aflatoxin B1 in *Escherichia coli* PQ37 strain	[64]
Leaves	3,5-*O*-Di-galloylquinic acid	Inhibitory effect against H_2_O_2_-induced genotoxicity, using the comet assay	[65]
		Gastrointestinal system	
Berry seeds	Hydroxybenzoic acid hexose Delphinidin-3-*O*-galactoside Delphinidin-3-*O*-glucoside Quercetin hexoside Delphinidin-3-*O*-rhamnoside Delphinidin rutinoside Delphinidin-3-(6 coumaroyl)-glucoside Petunidin-3-*O*-glucoside Petunidin diglucoside Petunidin malonylglucoside Petunidin-3-*O*-rutinoside Isorhamnetin-*O*-rhamnoside Malvidin-*O*-galactoside Malvidin-*O*-glucoside Peonidin diglucoside Petunidin methyl pentose	Anti-diarrhoeal in adult male Wistar rats after castor oil administration	[32]
Leaves	Not reported	Anti-diarrhoeal in Swiss albino mice of either sex weighing 20–30 g and aged 6–8 weeks, after castor oil administration	[69]
Berries	Not reported	Protective effect on gastric ulcer against ethanol, indomethacin, and pyloric ligation induced models in albino rats of Wistar strain weighing 150–200 g	[70]
Stems and seeds	Not reported	Protective effect on oral ulcer recovery process in white Spraque–Dawley rats weighing 250–300 g after punch to create a wound in the hard palate in the oral cavity	[71]
Berry seeds	Palmitic acid Stearic acid Oleic acid Linoleic acid Linolelaidic (*trans, trans*-C18:2) Arachidic acid	Protective effect on peptic ulcer against ethanol induced in adult male Wistar rats (weighing 220–240 g)	[72]
Berry seeds	Hydroxybenzoic acid hexose Delphinidin-3-*O*-galactoside Delphinidin-3-*O*-glucoside Quercetin hexoside Delphinidin-3-*O*-rhamnoside Delphinidin rutinoside Delphinidin-3-(6 coumaroyl)-glucoside Petunidin-3-*O*-glucoside Petunidin diglucoside Petunidin malonylglucoside Petunidin-3-*O*-rutinoside Isorhamnetin-*O*-rhamnoside Malvidin-*O*-galactoside Malvidin-*O*-glucoside Peonidin diglucoside Petunidin methyl pentose	Protective effect on acetic acid-induced ulcerative colitis in adult male Wistar rats (weighing 220–240 g)	[73]
Leaves	Not reported	Protective effect on acetic acid-induced ulcerative colitis in Wistar albino rats (weighing 250–300 g)	[74]
Leaves	Not reported	Protective effect on liver injury and fibrosis occurring in Wistar albino rats (weighing 250–300 g) with biliary obstruction by double ligatures with suture silk	[75]
Berries	Not reported	Decrease of reflux and dyspeptic scores as compared with the baseline, in double-blind randomized controlled clinical trial in adult aged from 18 to 60 years	[76]
Berries	Not reported	Decrease of the recurrence of symptoms in reflux patients after the discontinuance of proton pump inhibitors, in outpatient, double-blind, randomized, parallel treatment groups study	[77]
Leaves	Not reported	Decrease of the recurrent aphthous stomatitis in randomized, double blind, controlled before–after clinical trial	[78]
Leaves	Not reported	Decrease of recurrent aphthous stomatitis in a single-blind, placebo-controlled clinical trial	[79]
Aerial parts	Not reported	Upregulation of appetite related gene (ghrelin) and food intake in zebrafish	[80]
Aerial parts	Not reported	Spasmolytic: complete relaxation of spontaneous and K^+^ (80 mM)-induced contractions in isolated rabbit jejunum	[81]
		Cardiovascular system	
Leaves	Not reported	Anti-hypercholesterolemia by inhibition of 3-hydroxy-3-methylglutaryl coenzyme A reductase	[82]
Leaves	5,8-Dihydroxy-6,7,4′-trimethoxyflavone Quercetin-3-*O*-neohesperidoside, Quercetin-3-*O*-galactoside *trans*-1′,5′-5-(5-Carboxymethyl-2-oxocyclopentyl)-3*Z*-pentenyl-(6-*O*-galloyl) glucopyranoside, 3-Methoxy myricetin 7-*O*-β-l-rhamnopyranoside	Antiobesity effect on high fat diet induced male wistar albino obese rats	[83]
Aerial parts	Not reported	Vasodilator: Relaxation of phenylephrine (1 μM)- and K^+^ (80 mM)-induced contractions in isolated rabbit aorta	[81]
		Anti-hyperglycaemic	
Leaves	Not reported	Streptozotocin-induced diabetic female Swiss albino mice	[84]
Leaves	Not reported	Streptozotocin-induced diabetic 6-week-old male Albino Wistar rats	[85]
Not reported	Not reported	Inhibition of α-glucosidase from for baker’s yeast, rabbit liver, and rabbit small intestine	[86]
Aerial parts	Myrtucommulone B-E Usnone A Tectochrysine 2,5-Dihydroxy-4-methoxybenzophenone (cearoin) β-Sitosterol Sideroxylin Ursolic acid Corosolic acid Arjunolic acid Erythrodiol Oleanolic acid Betulin	Inhibition of α-glucosidase from *Saccharomyces* species	[50]
Aerial parts	Not reported	Streptozocin-induced type 1 diabetes mellitus in Sprague–Dawley male rats (weighing 225–250 g)	[87]
		Respiratory system	
Aerial parts	Not reported	Relaxant effect on carbachol- and K^+^ (80 mM)-induced contractions in isolated rabbit tracheal preparations	[81]
Berries	Not reported	Treatment of chronic rhinosinusitis in double-blinded randomized placebo-controlled trial	[88]
Leaves	Not reported	Inhibition of inflammation and fibrosis of lung parenchyma in both preventive and therapeutic methods in male albino rats weighting 180–200 g	[89]
		Nervous system	
Leaves	Not reported	Anxiolytic and muscle relaxant effect without anticonvulsant activities, hypnotic effects without effect on seizure threshold: Male NMRI mice subjected to open field, righting reflex, grip strength, and pentylentetrazole-induced seizure tests. Male Wistar rats used to evaluate the alterations in rapid eye movement (REM) and non-REM (NREM) sleep	[9]
Aerial parts	Not reported	Inhibition of acetylcholinesterse and butyrylcholinesterase	[90]
Aerial parts	Not reported	Antinociceptive activity using male albino mice weighing 25–30 g and the following tests: assessed using the hot plate and Writhing tests	[91]
		Skin	
Leaves	Not reported	Case report: Two patients with common warts	[92]
Leaves	Not reported	Treatment of dandruff: A double blinded randomized clinical trial comprised patients with dandruff aged 18–60 years visiting the dermatology out-patient clinic	[93]
		Genito-urinary system	
Berries	Not reported	A randomized, double-blind, placebo-controlled pilot study conducted on 30 women suffering from abnormal uterine bleeding-menometrorrhagia	[94]
		Longevity	
Leaves	Not reported	*Caenorhabditis elegans* used a model organism for longevity research and age-related diseases	[95]

The microorganisms reported in the Table are those that were used by the authors, such does not mean that samples have activity against all of the microorganisms.

**Table 2:** Phenols, flavonoids and anthocyanins in berry myrtle liqueurs and berry myrtle extracts.

Origin	Type of Extract	Identification/Quantification	Compounds	Reference
Italy (Sardinia)	- Traditional recipe for the preparation of the liqueur: maceration of fresh berries in ethanol:water (70:30) 960 mL for 40 days. - Lyophilized berries extracted by macerating berries in ethanol:water (70:30) for 40 days. - Fresh berries extracted by sonication for 1 h followed by maceration in ethanol:water (70:30) for one night.	HPLC-ESI-MS/HPLC-UV/VIS	*Anthocyanins*	[15]
			Delphinidin-3-*O*-glucoside	
			Cyanidin-3-*O*-glucoside	
			Petunidin 3-*O*-glucoside	
			Peonidin-3-*O*-glucoside	
			Malvidin-3-*O*-glucoside	
			Delphinidin-3-*O*-arabinoside	
			Petunidin-3-*O*-arabinoside	
			Malvidin-3-*O*-arabinoside	
	For quantitative determination: HPLC-UV/VIS using the internal standards cyanidin-3-*O*-galactopyranoside for anthocyans, and rutin (quercetin-3-*O*-rutinoside) for flavonoids		*Flavonoids*	
			Myricetin-3-*O*-galactoside	
			Myricetin-3-*O*-rhamnoside	
			Myricetin-3-*O*-arabinoside	
			Quercetin-3-*O*-glucoside	
			Quercetin-3-*O*-rhamnoside	
			Myricetin	
Italy (Sardinia)	- Berries extracted by maceration with ethanol, for six weeks, in the dark at 4 °C.	HPLC-MS/MS and HPLC-DAD	*Ethanol extract*	[110]
			Gallic acid derivatives—352.2	
			Gallic acid—111.5	
			Elagic acid—76.5	
			Other gallic acid derivatives—164.2	
			Anthocyanins—2195.0	
			Delphinidin-3-*O*-glucoside—494.8	
			Petunidin 3-*O*-glucoside—425.9	
			Malvidin-3-*O*-glucoside—840.9	
			Other anthocyanins—433.4	
			Flavonols—1492.8	
			Myricetin-3-*O*-galactoside—450.5	
			Myricetin-3-*O*-rhamnoside—441.2	
			Myricetin—342.2	
			Quercetin—36.2	
			Other flavonols—222.7	
			*Total*—4040.0 mg/mL	
	- Berries extracted by maceration with water, for six weeks, in the dark at 4 °C.		*Water extract*	
			Gallic acid derivatives—195.9	
			Gallic acid—76.0	
			Elagic acid—8.4	
			Other gallic acid derivatives—111.5	
			Anthocyanins—74.7	
			Delphinidin-3-*O*-glucoside—7.4	
			Petunidin-3-*O*-glucoside—11.2	
			Malvidin-3-*O*-glucoside—39.1	
			Other anthocyanins—17.0	
			Flavonols—103.0	
			Myricetin-3-*O*-galactoside- 23.4	
			Myricetin-3-*O*-rhamnoside- 52.9	
			Myricetin—	
			Quercetin—	
			Other flavonols—26.7	
	- Berries extracted by maceration with ethyl acetate, for six weeks, in the dark at 4 °C.		*Total*—373.6 (mg/mL)	
			*Ethyl acetate extract*	
	For quantitative determination: HPLC-DAD using calibration curves built with the method of external standard		Gallic acid derivatives—600.5	
			Gallic acid—361.7	
			Elagic acid—104.7	
			Other gallic acid derivatives—134.1	
			Anthocyanins—36.4	
			Delphinidin-3-*O*-glucoside—0.9	
			Petunidin 3-*O*-glucoside—1.7	
			Malvidin-3-*O*-glucoside—10.7	
			Other anthocyanins—1389.0	
			Flavonols—4.9	
			Myricetin-3-*O*-galactoside—216.9	
			Myricetin-3-*O*-rhamnoside—942.2	
			Myricetin—139.9	
			Quercetin—85.1	
			Other flavonols—26.7	
			*Total*—2025.9 (mg/L)	
Italy (Sardinia)	Obtained directly from producer and with known industrial processes	HPLC-MS/MS and HPLC-DAD	Hydroxybenzoic acids—18	[102]
			Gallic acid—12	
			Gallic acid derivatives—6	
			Flavanols—25	
			(+)-Catechin—25	
			Flavonols—124	
			Myricetin-3-*O*-arabinoside—51	
			Myricetin-3-*O*-galactoside—34	
			Myricetin-3-*O*-rhamnoside—3	
			Quercetin-3-*O*-glucoside—7	
			Quercetin-3-*O*-rhamnoside—6	
			Myricetin—20	
			Quercetin—3	
			Anthocyanins—110	
			Delphinidin-3-*O*-glucoside—20	
			Cyanidin-3-*O*-glucoside—5	
			Petunidin-3-*O*-glucoside—22	
			Peonidin-3-*O*-glucoside—5	
			Malvidin-3-*O*-glucoside—57	
			Anthocyanins arabinoside—11	
			*Total*—277 (mg/L)	
Italy (Sardinia)	Maceration in an ethanol–water mixture for four months. After separation of the berries of the macerates, the liqueurs were produced by adding sucrose and water to obtain a final percentage of 28% *v*/*v* (alcohol) and 32% *w*/*v* (sugar).	HPLC-MS/MS and HPLC-DAD	Hydroxybenzoic acids—408.2	[103]
			Gallic acid—294.2	
			Ellagic acid—55.8	
			Flavonols—58.1	
			Myricetin-3-*O*-galactoside—2.1	
			Myricetin-3-*O*-rhamnoside—23.0	
			Myricetin—25.6	
			Other flavonols—7.4	
			Anthocyanins—not detected	
			*Total*—466.4 (mg/L)	
Tunisia	Berries extracted by maceration with mixtures of ethanol/water (90:10—60:40) ethanol, for 40 days	HPLC/ESMS and HPLC/UV/Vis	Myricetin-3-*O*-arabinoside	[101]
			Myricetin-3-*O*-galactoside	
			Myricetin-3-*O*-rhamnoside	
			Quercetin-3-*O*-glucoside	
			Quercetin-3-*O*-rhamnoside	
			Myricetin	
			Quercetin	
			Kaempferol	
			Delphinidin-3-*O*-glucoside	
			Cyanidin-3-*O*-glucoside	
			Petunidin-3-*O*-glucoside	
			Delphinidin-3-*O*-arabinoside	
			Petunidin-3-*O*-glucoside	
			Peonidin-3-*O*-glucoside	
			Malvidin-3-*O*-glucoside	
			Petunidin-3-*O*-arabinoside	
			Malvidin-3-*O*-arabinoside	
Tunisia	Extraction with 70% MeOH for 24 h in a H_2_O bath shaker	HPLC/UV/Vis	*Dark blue fruits*	[106]
			Delphinidin-3-*O*-glucoside—172	
			Cyanidin-3-*O*-glucoside—25.2	
			Petunidin-3-*O*-glucoside—103.7	
			Delphinidin-3-*O*-arabinoside—28.3	
			Peonidin-3-*O*-glucoside—11.8	
			Malvidin-3-*O*-glucoside—257.6	
			Petunidin-3-*O*-arabinoside—18.8	
			Malvidin-3-*O*-arabinoside—8.6	
			*Total*—625.8 (mg malvidin-3-*O*-glucoside equivalent/100 mL)	
			*White fruits*	
			Delphinidin-3-*O*-glucoside—1.7	
			Cyanidin-3-*O*-glucoside—0.3	
			Petunidin-3-*O*-glucoside—0.9	
			Delphinidin-3-*O*-arabinoside—0.2	
			Peonidin-3-*O*-glucoside—0.2	
			Malvidin-3-*O*-glucoside—1.9	
			Petunidin-3-*O*-arabinoside—0.2	
			Malvidin-3-*O*-arabinoside—0.1	
			*Total*—5.4 (mg malvidin-3-*O*-glucoside equivalent/100 mL)	
Tunisia	Maceration	HPLC-DAD	*Whole fruit* (mg/g)	[107]
			Phenolic acids—1.03	
			Gallic acid—1.03	
			Hydrolysable tannins—0.69	
			Gallotannins—0.69	
			Flavonols—0.33	
			Quercetin-3-*O*-rutinoside—0.01	
			Myricetin-3-*O*-galactoside—0.08	
			Quercetin-3-*O*-galactoside—0.12	
			Myricetin-3-*O*-rhamnoside—0.07	
			Quercetin-3-*O*-rhamnoside—0.05	
			Anthocyanins—4.64	
			Delphinidin-3-*O*-glucoside—0.66	
			Cyanidin-3-*O*-glucoside—0.29	
			Petunidin-3-*O*-glucoside—0.89	
			Malvidin-3-*O*-glucoside—1.42	
			Petunidin-3-*O*-arabinoside—0.87	
			Malvidin-3-*O*-arabinoside—0.51	
			*Total*—6.69 mg/g	
			*Seed* (mg/g)	
			Phenolic acids—2.22	
			Gallic acid—2.22	
			Hydrolysable tannins—8.99	
			Gallotannins—8.99	
			Flavonols—	
			Quercetin-3-*O*-rutinoside—	
			Myricetin-3-*O*-galactoside—	
			Quercetin-3-*O*-galactoside—	
			Myricetin-3-*O*-rhamnoside—	
			Quercetin-3-*O*-rhamnoside—	
			Anthocyanins—	
			Delphinidin-3-*O*-glucoside—	
			Cyanidin-3-*O*-glucoside—	
			Petunidin-3-*O*-glucoside—	
			Malvidin-3-*O*-glucoside—	
			Petunidin-3-*O*-arabinoside—	
			Malvidin-3-*O*-arabinoside—	
			*Total*—11.11 mg/g	
			*Pericarp* (mg/g)	
			Phenolic acids—0.89	
			Gallic acid—0.89	
			Hydrolysable tannins—	
			Gallotannins—	
			Flavonols—0.33	
			Quercetin-3-*O*-rutinoside—0.01	
			Myricetin-3-*O*-galactoside—0.08	
			Quercetin-3-*O*-galactoside—0.12	
			Myricetin-3-*O*-rhamnoside—0.07	
			Quercetin-3-*O*-rhamnoside—0.05	
			Anthocyanins—3.74	
			Delphinidin-3-*O*-glucoside—0.66	
			Cyanidin-3-*O*-glucoside—0.19	
			Petunidin-3-*O*-glucoside—0.39	
			Malvidin-3-*O*-glucoside—1.12	
			Petunidin-3-*O*-arabinoside—0.87	
			Malvidin-3-*O*-arabinoside—0.51	
			*Total*—4.96 mg/g	
Tunisia	Sonication followed by maceration with methanol:water 1:1 (met/aq) Decoction using water (aq)	HPLC-DAD	*Leaves-September (aq—met/aq) (g/kg)*	[109]
			Gallic acid—16.90; 9.33	
			Delphinidin-3-*O*-glucoside—nd; nd	
			Myricetin-3-*O*-rhamnoside—18.26; 23.13	
			Quercetin-3-*O*-galactoside—0.24, 0.21	
			Quercetin-3-*O*-rutinoside—0.41; 0.45	
			Malvidin-3-*O*-glucoside—nd; nd	
			Myricetin—0.41; 0.41	
			Ellagic acid—5.15; 2.76	
			Quercetin—0.05; 0.09	
			Kaempferol—0.14; 0.14	
			*Total*—41.56; 36.52	
			*Leaves-December (aq—met/aq) (g/kg)*	
			Gallic acid—11.37; 0.79	
			Delphinidin-3-*O*-glucoside—nd; nd	
			Myricetin-3-*O*-rhamnoside—20.93; 18.95	
			Quercetin-3-*O*-galactoside—0.27, 0.29	
			Quercetin-3-*O*-rutinoside—0.51; 0.32	
			Malvidin-3-*O*-glucoside—nd; nd	
			Myricetin—4.20; 0.50	
			Ellagic acid—7.27; 3.52	
			Quercetin—0.16; 0.10	
			Kaempferol—0.22; 0.21	
			*Total*—44.93; 24.68	
			*Berries-September (aq—met/aq) (g/kg)*	
			Gallic acid—16.32; 5.00	
			Delphinidin-3-*O*-glucoside—nd; nd	
			Myricetin-3-*O*-rhamnoside—9.67; 9.80	
			Quercetin-3-*O*-galactoside—1.92, 2.05	
			Quercetin-3-*O*-rutinoside—together with quercetin-3-*O*-galactoside in both cases	
			Malvidin-3-*O*-glucoside—nd; nd	
			Myricetin—0.27; 0.31	
			Ellagic acid—19.10; 8.34	
			Quercetin—0.32; 0.28	
			Kaempferol—0.17; 0.11	
			*Total*—47.77; 25.89	
			*Berries-December (aq—met/aq) (g/kg)*	
			Gallic acid—4.54; 1.21	
			Delphinidin-3-*O*-glucoside—0.21; 0.16	
			Myricetin-3-*O*-rhamnoside—4.02; 5.69	
			Quercetin-3-*O*-galactoside—1.01, 1.22	
			Quercetin-3-*O*-rutinoside—together with quercetin-3-O-galactoside in both cases	
			Malvidin-3-*O*-glucoside—0.30; 0.32	
			Myricetin—0.31; 0.18	
			Ellagic acid—4.92; 2.99	
			Quercetin—0.04; 0.11	
			Kaempferol—0.09; 0.05	
			*Total*—15.44; 11.93	
			*Pericarps-December (aq—met/aq) (g/kg)*	
			Gallic acid—1.72; 0.24	
			Delphinidin-3-*O*-glucoside—0.22; 0.39	
			Myricetin-3-*O*-rhamnoside—3.10; 3.51	
			Quercetin-3-*O*-galactoside—0.40; 0.59	
			Quercetin-3-*O*-rutinoside—together with quercetin-3-O-galactoside in both cases	
			Malvidin-3-*O*-glucoside—0.42; 0.43	
			Myricetin—0.08; 0.08	
			Ellagic acid—0.69; 0.73	
			Quercetin—0.01; 0.02	
			Kaempferol—nd; 0.01	
			*Total*—6.64; 6.00	
			*Seeds-December (aq—met/aq) (g/kg)*	
			Gallic acid—16.62; 15.98	
			Delphinidin-3-*O*-glucoside—nd; nd	
			Myricetin-3-*O*-rhamnoside—2.25; 4.85	
			Quercetin-3-*O*-galactoside—0.33; 5.30	
			Quercetin-3-*O*-rutinoside—2.51; together with quercetin-3-*O*-galactoside in both cases	
			Malvidin-3-*O*-glucoside—nd; nd	
			Myricetin—1.90; 0.94	
			Ellagic acid—29.35; 21.18	
			Quercetin—0.18; 1.40	
			Kaempferol—0.19; 0.49	
			*Total*—53.33; 50.14	
Portugal	Sonication for 30 min, followed by maceration with water for 24 h, in the dark	HPLC–DAD–ESI–MS/MS	*Berries*	[112]
			Oenothein B	
			Galloyl-HHDP-glucose	
			Digalloyl HHDP-glucose	
			Quinic acid 3,5-di-*O*-gallate	
			Delphinidin-3-*O*-glucoside—1.33 mg/g	
			Cyanidin-3-*O*-glucoside—1.33 mg/g	
			Petunidin-3-*O*-glucoside—1.33 mg/g	
			Malvidin-3-*O*-monoglucoside—1.67 mg/g	
			Peonidin-3-*O*-monoglucoside—1.67 mg/g	
			Petunidin-3-*O*-pentoside—0.977 mg/g	
			Malvidin-3-*O*-pentoside—0.977 mg/g	
			Myricetin galactoside-gallate	
			Myricetin galactoside—0.00171 mg/g	
			Myricetin rhamnoside—0.00236 mg/g	
			Quercetin rhamnoside—0.000698 mg/g	
			*Leaves*	
			Myricetin galactoside-gallate—0.00261 mg/g	
			Myricetin galactoside—0.00261 mg/g	
			Myricetin rhamnoside—0.000255 mg/g	
			Myricetin—0.000075 mg/g	
			Quercetin galactoside-gallate—0.0136 mg/g	
Portugal	Liquid phase extraction (LPE) Supercritical fluid extraction (SFE)	HPLC–DAD–ESI–MS/MS	*Leaves and berries* (LPE)	[111]
			Gallic acid	
			Myricetin-3-*O*-rhamnoside V	
			Ellagic acid	
			Quercetin-*O*-rhamnoside	
			Myricetin	
			Kaempferol-*O*-rhamnoside	
			Quercetin	
			*Leaves* (SFE)	
			Myricetin-galactoside	
			Myricetin-rhamnoside	
			Quercetin-rhamnoside	
			*Berries* (SFE)	
			Myricetin-galactoside	
			Myricetin-3-*O*-rhamnoside	
			Quercetin-*O*-rhamnoside	
			Delphinidin-3-*O*-glucoside	
			Petunidin-3-*O*-glucoside	
			Malvidin-3-*O*-glucoside	
Italy (Sardinia)	Maceration in methanol	HPLC/UV	*Control*	[121]
			Phenolic acids (mg/g)	
			Gallic acid—0.17	
			Vanillic acid—0.10	
			Syringic acid—0.14	
			Ellagic acid—1.44	
			Flavonols/flavanols	
			Myricetin—1.11	
			Quercetin—0.20	
			Catechin—1.12	
			*Fermented homogenate*	
			Phenolic acids (mg/g)	
			Gallic acid—0.55	
			Vanillic acid—0.28	
			Syringic acid—0.28	
			Ellagic acid—2.78	
			Flavonols/flavanols	
			Myricetin—2.56	
			Quercetin—0.79	
			Catechin—1.26	
Tunisia	Maceration in water	HPLC–DAD–ESI–MS/MS	Hydroxybenzoic acid hexose	[32]
			Delphinidin-3-*O*-galactoside	
			Delphinidin-3-*O*-glucoside	
			Quercetin hexoside	
			Delphinidin-3-*O*-rhamnoside	
			Delphinidin rutinoside	
			Delphinidin-3-(6 coumaroyl)-glucoside	
			Petunidin-3-*O*-glucoside	
			Petunidin diglucoside	
			Petunidin malonylglucoside	
			Petunidin-3-*O*-rutinoside	
			Isorhamnetin-*O*-rhamnoside	
			Malvidin-*O*-galactoside	
			Malvidin-*O*-glucoside	
			Peonidin diglucoside	
			Petunidin methyl pentose	
Tunisia	Maceration in water	HPLC–DAD–ESI–MS/MS	Hydroxybenzoic acid hexose	[73]
			Delphinidin-3-*O*-galactoside	
			Delphinidin-3-*O*-glucoside	
			Quercetin hexoside	
			Delphinidin-3-*O*-rhamnoside	
			Delphinidin rutinoside	
			Delphinidin-3-(6 coumaroyl)-glucoside	
			Petunidin-3-*O*-glucoside	
			Petunidin diglucoside	
			Petunidin malonylglucoside	
			Petunidin-3-*O*-rutinoside	
			Isorhamnetin-*O*-rhamnoside	
			Malvidin-*O*-galactoside	
			Malvidin-*O*-glucoside	
			Peonidin diglucoside	
			Petunidin methyl pentose	
Italy	Flavoured sea salts	HPLC-DAD and 1H-NMR	*Phenols* (mg/100 g)	[122]
			Ellagic acid—11.7	
			Gallic acid—69.4	
			Myricetin—0.9	
			Myricetin-3-galactoside—7.3	
			Myricitrin—13.3	
			Quercetin-3-galactoside—0.9	
			Quercetin-3-glucoside	
			Quercitrin—1.1	
			Vitexin—0.7	

nd: not detected.

**Table 3:** Phenols, flavonoids, and acylphloroglucinols in leaf myrtle extracts.

Origin	Type of Extract	Identification/Quantification	Compounds	Reference
Italy	Hydroalcoholic extract, the remnant was fractionated by liquid–liquid extraction with ethyl acetate, and water residue	HPLC/MS and HPLC/DAD	*Hydroalcoholic extract*	[135]
			Galloyl derivatives (mg/mL)	
			Gallic acid—0.259	
			Mono, di-galloyl glucosides and ellagitannins—10.06	
			5-*O*-galloyl quinic acid—traces	
			3,5-*O*-galloyl quinic acid—0.64	
			Flavonols (mg/mL)	
			Myricitrin—0.91	
			Myricetin-3-*O*-galactoside—0.47	
			Myricetin-3-(6″-*O*-galloylgalactoside)—0.33	
			Myricetin glycosides—0.06	
			Quercitrin—0.02	
			*Ethyl acetate extract*	
			Galloyl derivatives (mg/mL)	
			Gallic acid—0.73	
			Mono, di-galloyl glucosides and ellagitannins—5.92	
			5-*O*-galloyl quinic acid—traces	
			3,5-*O*-galloyl quinic acid—1.49	
			Flavonols (mg/mL)	
			Myricitrin—2.83	
			Myricetin-3-*O*-galactoside—1.54	
			Myricetin-3-(6″-O-galloylgalactoside)—1.07	
			Myricetin glycosides—0.23	
			Quercitrin—0.07	
			*Aqueous residue*	
			Galloyl derivatives (mg/mL)	
			Mono, di-galloyl glucosides and ellagitannins—0.30	
			Flavonols (mg/mL)	
			Myricitrin—0.001	
Tunisia	Acid hydrolysis with HCl 1 M, sonication, and decoction with water	HPLC/UV/Vis	*Leaf*	[125]
			Phenolic acids—1.40 (mg/g)	
			Gallic acid—1.05	
			Caffeic acid—0.08	
			Syringic acid—0.08	
			Vanillic acid—0.04	
			Ferulic acid—0.05	
			Hydrolysable tannins—8.90 (mg/g)	
			Gallotannins—8.75	
			Flavonoids—0.91 (mg/g)	
			Quercetin-3-rutinoside—	
			Myricetin-3-*O*-galactoside—0.23	
			Quercetin-3-galactoside—0.13	
			Myricetin-3-*O*-rahmnoside—0.05	
			Quercetin-3-*O*-rahmnoside—0.29	
			Myricetin—0.10	
			Quercetin—0.11	
			Catechin—traces	
			Unknown—0.15 (mg/g)	
			*Total*—11.21 (mg/g)	
			*Stem*	
			Phenolic acids—1.17 (mg/g)	
			Gallic acid—1.02	
			Caffeic acid—	
			Syringic acid—0.08	
			Vanillic acid—0.02	
			Ferulic acid—0.05	
			Hydrolysable tannins—traces (mg/g)	
			Gallotannins—traces	
			Flavonoids—1.86 (mg/g)	
			Quercetin-3-rutinoside—0.08	
			Myricetin-3-*O*-galactoside—0.11	
			Quercetin-3-galactoside—0.12	
			Myricetin-3-*O*-rahmnoside—0.15	
			Quercetin-3-*O*-rahmnoside—0.09	
			Myricetin—0.19	
			Quercetin—	
			Catechin—1.12	
			Unknown—(mg/g)	
			*Total*—3.03 (mg/g)	
			*Flower*	
			Phenolic acids—2.34 (mg/g)	
			Gallic acid—2.34	
			Caffeic acid—	
			Syringic acid—	
			Vanillic acid—	
			Ferulic acid—	
			Hydrolysable tannins—3.50 (mg/g)	
			Gallotannins—3.50	
			Flavonoids—traces (mg/g)	
			Quercetin-3-rutinoside–	
			Myricetin-3-*O*-galactoside—traces	
			Quercetin-3-galactoside—	
			Myricetin-3-*O*-rahmnoside—traces	
			Quercetin-3-*O*-rahmnoside—	
			Myricetin—	
			Quercetin—	
			Catechin—traces	
			Unknown—0.19 (mg/g)	
			*Total*—6.02 (mg/g)	
Algeria	- Maceration in hydroalcoholic solution (50:50) - Hydroalcoholic extract (50:50) irradiated by microwaves (700 w), for 1 min	HPLC-DAD	*Microwave assisted extraction*	[147]
			Galloylquinic acid—7.33 GAE mg/g	
			Gallic acid—3.53 mg/g	
			Myricetin-3-*O*-galactoside—2.38 mg MRE/g	
			Myricetin-3-*O*-rhamnoside (MR)—12.26 mg/g	
			Ellagic acid—0.84 mg MRE/g	
			*Maceration*	
			Galloylquinic acid—7.66 GAE mg/g	
			Gallic acid—3.31 mg/g	
			Myricetin-3-*O*-galactoside—2.37 mg MRE/g	
			Myricetin-3-*O*-rhamnoside (MR)—11.78 mg/g	
			Ellagic acid—0.88 mg MRE/g	
Algeria	Microwave-assisted extraction Ultrasound-assisted extraction Maceration	HPLC-DAD	Galloylquinic acid	[138]
			Gallic acid	
			Gallotannin	
			Myricetin-3-*O*-galactoside	
			Digalloylquinic acid	
			Trigalloylquinic HHDD-glucose	
			Myricetin galloylgalactoside	
			Myricetin-3-*O*-rhamnoside	
			Quercetin-3-*O*-rhamnoside	
Iran	Maceration with methanol	HPLC	*Spring* (mg/100 g)	[123]
			Gallic acid—5.32	
			Chlorogenic acid—2.89	
			*p*-Coumaric acid—11.73	
			Ferulic acid—85.56	
			Rutin—10.87	
			Luteolin—2.21	
			Quercetin—5.23	
			Apigenin—7.45	
			*Summer* (mg/100 g)	
			Gallic acid—6.72	
			Chlorogenic acid—3.79	
			*p*-Coumaric acid—14.13	
			Ferulic acid—94.71	
			Rutin—16.48	
			Luteolin—1.72	
			Quercetin—6.41	
			Apigenin—8.70	
			*Fall* (mg/100 g)	
			Gallic acid—18.79	
			Chlorogenic acid—3.70	
			*p*-Coumaric acid—15.24	
			Ferulic acid—168.89	
			Rutin—35.38	
			Luteolin—3.40	
			Quercetin—5.70	
			Apigenin—10.07	
Tunisia	Decoction in water: 5, 10, and 15 min of heating	HPLC-UV/Vis	5 min (μmol/g)	[141]
			Gallic acid—6.47	
			Caffeic acid—0.71	
			Syringic acid—0.18	
			Ferulic acid—0.29	
			Myricetin-3-*O*- galactoside—0.59	
			Myricetin-3-*O*-rhamnoside—0.71	
			Myricetin-3-*O*-arabinoside—0.12	
			Quercetin-3-*O*-galactoside—5.35	
			Quercetin-3-*O*-rhamnoside—0.29	
			Myricetin—3.00	
			Quercetin—1.59	
			*Total*—19.28	
			*Phenolic acids*—7.64	
			*Flavonol glycosides*—7.05	
			*Flavonols—*4.58	
			10 min (μmol/g)	
			Gallic acid—8.23	
			Caffeic acid—0.88	
			Syringic acid—0.29	
			Ferulic acid—0.41	
			Myricetin-3-*O*-galactoside—0.76	
			Myricetin-3-*O*-rhamnoside—0.82	
			Myricetin-3-*O*-arabinoside—0.18	
			Quercetin-3-*O*-galactoside—6.64	
			Quercetin-3-*O*-rhamnoside—0.29	
			Myricetin—3.82	
			Quercetin—2.41	
			*Total*—24.75	
			*Phenolic acids*—9.82	
			*Flavonol glycosides*—8.70	
			*Flavonols—*6.23	
			15 min (μmol/g)	
			Gallic acid—11.82	
			Caffeic acid—1.41	
			Syringic acid—0.53	
			Ferulic acid—0.53	
			Myricetin-3-*O*-galactoside—1.06	
			Myricetin-3-*O*-rhamnoside—1.18	
			Myricetin-3-*O*-arabinoside—0.24	
			Quercetin-3-*O*-galactoside—9.11	
			Quercetin-3-*O*-rhamnoside—0.53	
			Myricetin—5.00	
			Quercetin—3.59	
			*Total*—34.98	
			*Phenolic acids*—14.28	
			*Flavonol glycosides*—12.11	
			*Flavonols—*8.58	
Not reported	Not reported	Single-crystal X-ray diffraction 1H-NMR, high resolution electrospray ionization mass spectrometry, and Heteronuclear multiple-bond correlation spectroscopy	Myrtucommuacetalone	[143]
			Myrtucommulone M	
			Myricetin	
			Isousnic acid	
			Growth regulator G3 factor	
			Myrtucommulone E	
Italy	Aqueous	HPLC/DAD/ESI-MS methods	Aqueous (2009–2010) fresh material (mmol/L)	[145]
			HHDP glucose—0.151–nd	
			Monogalloyl-glucose—0.102–nd	
			Galloylquinic acid—nd–0.169	
			Gallic acid—2.712–4.232	
			Gallotannin *m*/*z* 801—0.545–nd	
			Gallotannin *m*/*z* 429—nd–nd	
			Gallotannin *m*/*z* 633—0.249–0.300	
			Gallotannin *m*/*z* 633—0.134–nd	
			Gallotannin *m*/*z* 801—0.338–0.335	
			Gallotannin *m*/*z* 633—0.484–nd	
			Gallotannin *m*/*z* 1583—nd–nd	
			Ellagitannin *m*/*z* 933—0.132–0.067	
			Galloylquinic acid—0.619–0.355	
			Gallotannin *m*/*z* 1565—3.378–0.400	
			Gallotannin *m*/*z* 1567—2.403–2.344	
			Gallotannin *m*/*z* 935—1.075–nd	
			Digalloylquinic acid—nd–0.711	
			Trigalloyl HHDP-glucose—nd–0.423	
			Ellagitannin *m*/*z* 953—0.261–0.551	
			Gallotannin *m*/*z* 783—5.824–nd	
			Ellagitannin *m*/*z* 1253—nd–0.254	
			Ellagitannin *m*/*z* 953—0.240–nd	
			Ellagitannin *m*/*z* 1085—0.336–0.239	
			Myricetin galloylgalactoside—0.297–0.429	
			Myricetin 3-*O*-galactoside—0.274–0.754	
			Myricetin 3-*O*-rhamnoside—1.326–1.945	
			Ellagic acid—0.266–0.757	
			Quercetin-3-O-rhamnoside—1.326–1.945	
			*Total polyphenols*—*21.148*–*14.266*	
			Aqueous—hydroalcoholic (2010) dried material (mmol/L)	
			HHDP glucose—nd–nd	
			Monogalloyl-glucose—nd–nd	
			Galloylquinic acid—0.208–0.144	
			Gallic acid—4.489–0.268	
			Gallotannin *m*/*z* 801—nd–nd	
			Gallotannin *m*/*z* 429—nd–0.144	
			Gallotannin *m*/*z* 633—0.435–0.265	
			Gallotannin *m*/*z* 633—nd–nd	
			Gallotannin *m*/*z* 801—0.397–0.154	
			Gallotannin *m*/*z* 633—nd–nd	
			Gallotannin *m*/*z* 1583—nd–7.050	
			Ellagitannin *m*/*z* 933—0.189–nd	
			Galloylquinic acid—0.386–0.374	
			Gallotannin *m*/*z* 1565—0.606–0.495	
			Gallotannin *m*/*z* 1567—2.968–2.678	
			Gallotannin *m*/*z* 935—0.286–nd	
			Digalloylquinic acid—0.559–1.574	
			Trigalloyl HHDP-glucose—nd–nd	
			Ellagitannin *m*/*z* 953—nd–nd	
			Gallotannin *m*/*z* 783—nd–nd	
			Ellagitannin *m*/*z* 1253—0.407–0.416	
			Ellagitannin *m*/*z* 953—0.794–nd	
			Ellagitannin *m*/*z* 1085—0.276–nd	
			Myricetin galloylgalactoside—0.300–0.486	
			Myricetin 3-*O*-galactoside—0.620–1.084	
			Myricetin 3-*O*-rhamnoside—1.392–2.526	
			Ellagic acid—0.918–0.612	
			Quercetin-3-O-rhamnoside—nd–0.038	
			*Total polyphenols*—*15.229*–*18.308*	
Japan	Aqueous acetone 70%	Preparative chromatography and comparison of spectroscopy data with those previously reported	Oenothein B	[122]
			Eugeniflorin	
			Tellimagrandin I	
			Tellimagrandin II	
			Gallic acid	
			Quinic acid 3,5-di-*O*-gallate	
			Myricetin-3-*O*-β-d-xyloside	
			Myricetin-3-*O*-β-d-galactoside	
			Myricetin-3-*O*-β-d-galactoside-6″-*O*-gallate	
			Myricetin-3-*O*-β-l-rhamnoside	

nd: not detected.

**Table 4:** Phenols and flavonoids in Saharan myrtle extracts.

Origin	Type of Extract	Identification/Quantification	Compounds	Reference
Algeria (Sahara)	Infusion and decoction	UHPLC-PDA-HRMS, NMR and HPLC-UV-PDA	Roseoside	[20]
			2-Hydroxy-1,8-cineole-β-d-glucopyranoside	
			2-Hydroxy-1,8-cineole 2-*O*-α-l-arabinofuranosyl (1→6)-β-d-glucopyranoside	
			3,4,5-Tri-*O*-galloyl-quinic acid	
			Myricetin-3-*O*-β-d(6″-galloyl)glucopyranoside	
			Isomyricitrin	
			1,2,3,6-Tetra-*O*-galloyl glucose	
			Myricitrin	
			Quercetin-3-*O*-β-d-(6″-galloyl)glucopyranoside	
			Myricetin-3-*O*-β-xyloside	
			Isoquercitrin	
			3-Oxo-α-ionol-9-*O*-β-d-glucopyranoside	
			Myricetin	
			Quercitrin	
Algeria (Sahara)	Decoction water Fractionation: ethyl acetate and butanol	Liquid chromatography with diode array detection, coupled to mass spectrometry (ion trap) with electrospray ionization (HPLC-DAD−ESI/MS*n*)	Crude aqueous extract (mg/g)	[22]
			Galloyl-HHDP-glucoside—nd	
			Galloyl-HHDP-glucoside—29.0	
			Digalloylquinic acid—nd	
			Digalloyl-HHDP-glucoside—14.5	
			Trigalloylglucoside—3.0	
			Rugosin B—5.4	
			Digalloyl-HHDP-glucoside—8.9	
			Trigalloylquinic acid—11.2	
			Trigalloylglucoside—nd	
			Digalloyl-HHDP-glucoside—7.22	
			Gallocatechin-gallate-dimer—10.9	
			Valoneic acid dilactone—nd	
			Myricetin-hhexosyl-gallate—13.4	
			Trigalloyl-HHDP-glucoside—4.63	
			Myricetin-3-*O*-glucoside—8.48	
			Tetragalloylglucose—nd	
			Rugosin A—4.9	
			Tetragalloylglucose—4.02	
			Quercetin-hexoxyl-gallate—1.88	
			Myricetin-3-*O*-rhamnoside—11.3	
			Quercetin-3-*O*-glucoside—1.64	
			Ellagic acid—nd	
			Kaempferol-hexosyl-gallate—4.3	
			Kaempferol-3-*O*-glucoside—nd	
			Quercetin-3-*O*-rhamnoside—nd	
			Myricetin—nd	
			Myricetin-coumaroylhexoside—nd	
			Total hydrosable tannins—93 (mg/g)	
			Total phenolic acids—	
			Total flavonoids—45	
			Total phenolic compounds—138	
			*Ethyl acetate fraction* (mg/g)	
			Galloyl-HHDP-glucoside—nd	
			Galloyl-HHDP-glucoside—nd	
			Digalloylquinic acid—6.41	
			Digalloyl-HHDP-glucoside—19.28	
			Trigalloylglucoside—7.1	
			Rugosin B—7.0	
			Digalloyl-HHDP-glucoside—20.72	
			Trigalloylquinic acid—22.2	
			Trigalloylglucoside—12.3	
			Digalloyl-HHDP-glucoside—11.1	
			Gallocatechin-gallate-dimer—35.6	
			Valoneic acid dilactone—9.5	
			Myricetin-hhexosyl-gallate—37.0	
			Trigalloyl-HHDP-glucoside—17.1	
			Myricetin-3-*O*-glucoside—19.88	
			Tetragalloylglucose—16.3	
			Rugosin A—9.7	
			Tetragalloylglucose—13.2	
			Quercetin-hexoxyl-gallate—10.54	
			Myricetin-3-*O*-rhamnoside—85.75	
			Quercetin-3-*O*-glucoside—3.1	
			Ellagic acid—27.1	
			Kaempferol-hexosyl-gallate—8.34	
			Kaempferol-3-*O*-glucoside—3.3	
			Quercetin-3-*O*-rhamnoside—3.2	
			Myricetin—5.5	
			Myricetin-coumaroylhexoside—2.12	
			Total hydrosable tannins—172 (mg/g)	
			Total phenolic acids—27.08	
			Total flavonoids—200	
			Total phenolic compounds—398	
			*Butanol fraction* (mg/g)	
			Galloyl-HHDP-glucoside—10.01	
			Galloyl-HHDP-glucoside—17.4	
			Digalloylquinic acid—14.3	
			Digalloyl-HHDP-glucoside—26.7	
			Trigalloylglucoside—6.18	
			Rugosin B—9.4	
			Digalloyl-HHDP-glucoside—16.9	
			Trigalloylquinic acid—31.3	
			Trigalloylglucoside—12.2	
			Digalloyl-HHDP-glucoside—6.6	
			Gallocatechin-gallate-dimer—13.2	
			Valoneic acid dilactone—8.47	
			Myricetin-hhexosyl-gallate—17.8	
			Trigalloyl-HHDP-glucoside—nd	
			Myricetin-3-*O*-glucoside—23.6	
			Tetragalloylglucose—nd	
			Rugosin A—7.8	
			Tetragalloylglucose—nd	
			Quercetin-hexoxyl-gallate—1.99	
			Myricetin-3-*O*-rhamnoside—12.4	
			Quercetin-3-*O*-glucoside—2.18	
			Ellagic acid—6.4	
			Kaempferol-hexosyl-gallate—nd	
			Kaempferol-3-*O*-glucoside—nd	
			Quercetin-3-*O*-rhamnoside—nd	
			Myricetin—nd	
			Myricetin-coumaroylhexoside—nd	
			Total hydrosable tannins—167.5 (mg/g)	
			Total phenolic acids—6.4	
			Total flavonoids—62.9	
			Total phenolic compounds—236.8	

nd: not detected
